# Multicellular gene network analysis identifies a macrophage-related gene signature predictive of therapeutic response and prognosis of gliomas

**DOI:** 10.1186/s12967-019-1908-1

**Published:** 2019-05-16

**Authors:** Xiaoqiang Sun, Xiaoping Liu, Mengxue Xia, Yongzhao Shao, Xiaohua Douglas Zhang

**Affiliations:** 1 Department of Medical Informatics, Zhong-shan School of Medicine, Sun Yat-Sen University, Guangzhou, 510089 China; 2 School of Mathematics, Sun Yat-Sen University, Guangzhou, 510089 China; 3 School of Mathematics and Statistics, Shandong University at Weihai, Weihai, China; 4 NYU School of Medicine, NYU Langone Health, New York University, New York, NY 10016 USA; 5 Faculty of Health Sciences, University of Macau, Taipa, Macau China

**Keywords:** Multicellular gene network, Macrophages, Prognostic signature, Drug resistance, Glioma, Biomarker

## Abstract

**Background:**

The tumor-associated microenvironment plays important roles in tumor progression and drug resistance. However, systematic investigations of macrophage–tumor cell interactions to identify novel macrophage-related gene signatures in gliomas for predicting patient prognoses and responses to targeted therapies are lacking.

**Methods:**

We developed a multicellular gene network approach to investigating the prognostic role of macrophage–tumor cell interactions in tumor progression and drug resistance in gliomas. Multicellular gene networks connecting macrophages and tumor cells were constructed from re-grouped drug-sensitive and drug-resistant samples of RNA-seq data in mice gliomas treated with BLZ945 (a CSF1R inhibitor). Subsequently, a differential network-based COX regression model was built to identify the risk signature using a cohort of 310 glioma samples from the Chinese Glioma Genome Atlas database. A large independent validation set of 690 glioma samples from The Cancer Genome Atlas database was used to test the prognostic significance and accuracy of the gene signature in predicting prognosis and targeted therapeutic response of glioma patients.

**Results:**

A macrophage-related gene signature was developed consisting of twelve genes (ANPEP, DPP4, PRRG1, GPNMB, TMEM26, PXDN, CDH6, SCN3A, SEMA6B, CCDC37, FANCA, NETO2), which was tested in the independent validation set to examine its prognostic significance and accuracy. The generation of 1000 random gene signatures by a bootstrapping scheme justified the non-random nature of the macrophage-related gene signature. Moreover, the discovered gene signature was verified to be predictive of the sensitivity or resistance of glioma patients to molecularly targeted therapeutics and outperformed other existing gene signatures. Additionally, the macrophage-related gene signature was an independent and the strongest prognostic factor when adjusted for clinicopathologic risk factors and other existing gene signatures.

**Conclusion:**

The multicellular gene network approach developed herein indicates profound roles of the macrophage-mediated tumor microenvironment in the progression and drug resistance of gliomas. The identified macrophage-related gene signature has good prognostic value for predicting resistance to targeted therapeutics and survival of glioma patients, implying that combining current targeted therapies with new macrophage-targeted therapy may be beneficial for the long-term treatment outcomes of glioma patients.

**Electronic supplementary material:**

The online version of this article (10.1186/s12967-019-1908-1) contains supplementary material, which is available to authorized users.

## Background

Gliomas are among the most malignant cancers, commonly inducing profound and progressive disability and causing death in most cases [1]. Although many cancer cell-targeted therapeutic agents have been developed, intrinsic or acquired resistance to such therapies often emerges during long-term treatment [2]. Therefore, the preexisting or newly developed tolerance of cancer cells to molecularly targeted therapeutic drugs is a main cause of the eventual failure of most existing targeted therapies.

Several forms of cancer cell-intrinsic mechanisms of drug resistance have been revealed, including genetic/epigenetic mechanisms [3], posttranslational mechanisms [4–6], cellular mechanisms [7, 8], and metabolic mechanisms [9]. Recently, an increasing number of experiments indicated that the tumor microenvironment may play important roles in cancer progression and drug resistance [10]. Therefore, uncovering the intercellular interactions between cancer cells and microenvironmental cells is crucial for identifying effective biomarkers for predicting drug resistance and cancer progression, as well as understanding the mechanisms of acquired resistance and prioritizing potential drug targets. Importantly, therapies targeting the tumor microenvironment have been proposed as a promising approach for treating cancers, including gliomas [11, 12]. Several macrophage-targeted therapies for gliomas have been developed [10], further highlighting the importance of tumor–microenvironment interactions in determining glioma outcome.

Systems biology approaches are powerful for quantitatively studying various forms of drug resistance at multiple scales, which can provide insights into underlying mechanisms and experimentally testable predictions [13]. Computational methods have also been used to identify prognostic biomarkers and predict drug resistance. However, conventional methods for glioma biomarker identification often focus on individual cell types or molecules rather than on the interactions between different cell types and molecules. Exploring the interactions between tumor cells and microenvironmental cells, such as macrophages, from a network view can more insightfully help understand the mechanisms that underlie cancer progression and drug resistance and discover more robust and accurate biomarkers [14].

In this study, we developed a multicellular gene network-based approach to investigating intercellular gene associations between tumor cells (TCs) and tumor-associated macrophages (TAMs) and to identify biomarkers of prognosis and drug resistance in gliomas. We used RNA-seq data from mice bearing gliomas treated with BLZ945 to construct drug-sensitive and drug-resistant multicellular gene networks. Based on the differential network, a macrophage-related gene signature was discovered using a dataset of glioma patients from the Chinese Glioma Genome Atlas (CGGA) database. An independent dataset from The Cancer Genome Atlas (TCGA) database was used for validation. Time-dependent receiver operator characteristics (ROC) analysis was used to assess the prognostic significance and accuracy of the gene signature for predicting survival of glioma patients. Moreover, the macrophage-related gene signature was found to be predictive of the targeted therapy outcome in glioma patients and outperformed existing gene signatures including conventional EGFR signature [15, 16] and an immune-related gene signature [17], which were verified using the validation dataset. Moreover, we showed that the network perturbation analysis improved the model-building process beyond the conventional methods of identifying signature genes based on differential expression.

## Methods

### Preclinical RNA-seq data analysis

RNA-seq data (FPKM) of TCs and TAMs in mice gliomas were downloaded from the Gene Expression Omnibus (GEO) website (https://www.ncbi.nlm.nih.gov/geo/) under accession number GSE69104. The preclinical experiment [10] investigated the role of TAMs in glioma immunotherapy with BLZ945, a CSF1R inhibitor, where all TC-TAM paired samples were divided into 3 groups, i.e., Vehicle (Veh, 5 samples), Endpoint (EP, 6 samples, i.e., drug-sensitive), and Rebound (Reb, 4 samples, i.e., drug-resistant) tumors (Additional file 1: Table S1).

As described in detail in Additional file 1: Text S1, the expression level of each gene in the drug-resistant and drug-sensitive samples were normalized to their mean values in the vehicle samples. We selected significantly differentially expressed genes (DEGs) between the drug-resistant samples (Reb) and the drug-sensitive samples (Ep) for both TCs and TAMs by calculating the fold changes (FCs) and false discovery rate (FDR)-adjusted *p* values using *t* test. A gene with |FC| > 1.5 and adjusted *p* value less than 0.05 was considered as a DEG, resulting in a list of 1141 DEGs.

### Multicellular gene network construction and differential network analysis

The top 50 DEGs with the largest absolute fold change value in each type of cells were selected for gene network construction. We built multicellular gene association networks between TAMs and TCs to investigate the changes between these cell types during the emergence of drug resistance from a network perspective. We classified the above DEGs into TC-specific and TAM-specific DEGs and computed the Pearson correlation coefficients (PCCs) for each pair of these DEGs. An edge is added to the TC-TAM gene network if the corresponding |PCC| > 0.95 and *p* value < 0.05. In this way, we built two-types of edges, including ‘intracellular edges’ within TCs or TAMs and ‘intercellular edges’ between TCs and TAMs, to construct the multicellular gene network.

**Fig. 1:**
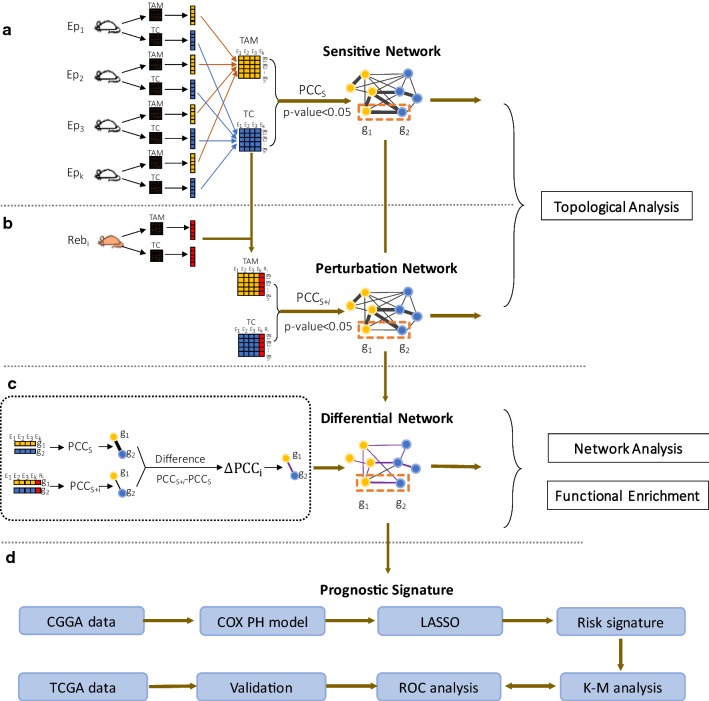
Schematic illustration of the multicellular gene network-based identification of risk signatures. **a** The correlation (e.g., PCCs) of each pair of differential genes in TAMs and TCs of the Endpoint samples were computed to construct a sensitive multicellular network. **b** Each single Rebound sample was added to the Endpoint samples to construct a sample-specific resistance multicellular network (Additional file 1: Fig S2–S6). **c** A robust differential network was constructed using the differences between correlation coefficients of gene pairs in the sensitive and perturbation networks. Topological analysis, signature gene analysis and gene enrichment analysis were used to analyze the networks. **d** Based on the differential network, we used the COX PH model and LASSO method to select prognostic genes and define a risk signature. The clinical information and RNA-seq expression data in the CGGA and TCGA databases were used as learning and validation sets, respectively

Based on the work of one of our authors [18], we developed a network perturbation analysis technique for multicellular gene networks. As described in detail in Text S1, first we used the reference samples (N = 6) in the Ep group to construct a sensitive network (Fig. 1a) for pairs of correlated DEGs in TCs and TAMs (|PCC| > 0.95 and *p* value < 0.05). We then added each single drug-resistant sample (Reb_i_, *i* = 1, 2, 3, 4) to the reference samples in the Ep group to construct 4 sets of sample-specific perturbed samples, respectively (Additional file 1: Table S1). We used each set of perturbed samples (N = 7) to construct perturbation networks (Fig. 1b). The addition of each single Reb sample is the cause of differences between the sensitive and perturbation networks. If the gene expression profile in the added sample, Reb_i_, was similar to that in the Ep samples, the perturbation of the PCC was negligible. However, if some gene expression levels were remarkably different between the single Reb sample and the Ep samples, significant changes in the PCCs of certain gene pairs were induced upon the addition of Reb_i_ to the reference samples.

Based on such network perturbation analysis (Fig. 1c), the robust significantly different PCCs (ΔPCC, see Text S1) of gene pairs were chosen to construct a differential network. Specifically, we selected significantly different edges, represented by ΔPCC, by setting a threshold of |ΔPCC| > 0.05 for each perturbation network versus the sensitive network. If a gene-pair was differentially correlated in at least 3 perturbation networks compared to the sensitive network, this edge was selected as a robust differential edge. We defined three types of edges in the differential network: correlation-gained edges (ΔPCC > 0), correlation-lost edges (ΔPCC < 0), and correlation-invariant edges (ΔPCC = 0). The differential network reflected the changes in both intracellular and intercellular edges in the multicellular gene networks of mice with distinct therapeutic responses to the CSF1R inhibition.

### Functional enrichment analysis

The functional enrichment of genes in the differential network was analyzed using the newest version of Metascape (http://metascape.org), a gene annotation and enrichment analysis database that integrates ontology sources, including the KEGG Pathway, GO Biological Processes, Reactome Gene Sets, Canonical Pathways and CORUM databases. The whole genome was used as the enrichment background. Terms of pathways or processes with a *p* value < 0.01 (accumulative hypergeometric test), a minimum of 3 genes, and an enrichment factor > 1.5 were collected and grouped into clusters based on their membership similarities.

### Prognostic signature identification and validation

The differential network might capture robust topological differences between the CSF1R inhibitor-sensitive network and inhibitor-resistant networks and reflect potential changes in the gene interactions across TCs and TAMs during the acquisition and development of drug resistance. It is therefore reasonable to speculate that the genes in the differential network play critical roles in promoting tumor growth, even under drug pressure. We hypothesized that the expression levels of genes in the differential network are associated with the survival outcomes of glioma patients. As such, we developed a differential network-based signature identification method to select prognostic biomarkers for glioma patients. We collected the clinical information and RNA-seq gene expression data from glioma patients in the CGGA database (http://www.cgga.org.cn/) and TCGA database (https://cancergenome.nih.gov/). The names of genes in the differential network were mapped to those of genes in homo sapiens, resulting in a list of 29 candidate genes. By matching both patient sample IDs and gene names from the clinical information and the gene expression data, a learning set of 310 samples from CGGA dataset and an independent validation set of 690 samples from TCGA dataset were created for prognostic signature identification, validation and further analysis. The details of the sample information were listed in Additional file 2: Table S2.

We used a multivariate COX proportional hazards (PH) model [19] and LASSO regression method [20] to select prognostic genes from the differential gene association network (see details in Text S1). A tenfold cross-validation was performed to select the optimal values of the tuning parameter for minimizing the mean cross-validation error.

The regression coefficients at the optimal tuning parameter were computed as risk coefficients, which were used to formularize a risk signature. The same risk signature was used to compute the risk scores (RSs) for patients in the independent validation set. The patients in each dataset were classified into a high-risk group and a low-risk group according to the optimal cutoff value using the ROC method. The Kaplan–Meier (K–M) curves for patients in the high- and low-risk groups were analyzed, and the statistical significance of the difference was assessed using the two-sided log-rank test. Time-dependent ROC analysis [21] was further conducted to evaluate the prognostic accuracy of the above RSs with respect to the 3- and 5-year survival predictions of patients in both the learning and independent validation sets.

### Prediction of response to targeted therapeutics

The survival and gene expression data from glioma patients who received targeted therapies in the independent set were extracted to evaluate the predictive effectiveness of the macrophage-related gene signature for classifying patients into drug-sensitive and drug-resistant groups. We used the observed 3- or 5-year survival status to substitute for the latent drug-resistance status in these patients. Specifically, the 3- or 5-year survival status (alive or dead) was defined as the outcome (sensitive or resistant) of targeted drug treatment. The above risk signature was used to classify each patient into a sensitive group (i.e., low-risk group) or a resistant group (i.e., high-risk group) according to the optimal cutoff value of the RS using the ROC method. To further compare the powers of different gene signatures to predict responses to targeted therapy, we calculated the area under the curves (AUCs) of the time-dependent ROCs to assess their accuracies.

### Comparison with other methods and other related signatures

We compared the macrophage-related gene signature produced from the above multicellular gene network perturbation method with other commonly used methods, including LASSO Cox regression model [22] and correlation network-based biomarker identification method without network perturbation. First, a LASSO Cox signature was trained from the full list of 1141 DEGs based on the CGGA set. In addition, a weighted correlation network (without perturbation) was constructed by calculating the pairwise Person correlation coefficients (PCCs) across all pairs of DEGs within both TCs and TAMs for the full set of Veh, Reb and Ep samples (30 samples). Based on the ranking of node strength score (defined as the sum of the absolute values of correlation coefficients of the node with other nodes), we selected the top ranked 12 DEGs as a gene set for defining a prognostic signature using the CGGA dataset.

Moreover, to assess whether the macrophage-related gene signature was independently correlated with the prognosis of glioma patients, we conducted univariate and multivariate COX regression analyses of clinicopathological factors and available gene signatures. Clinicopathological information, including age, gender and grade, was available for glioma patients in both the learning and validation sets. We also included the following gene signatures in the multivariate COX regression analyses: signature 1—the macrophage-related gene signature newly proposed in this study; signature 2—an EGFR gene signature studied by many groups [15, 16]; and an immune-related gene signature for predicting the prognosis of glioma patients, i.e., signature 3—the Cheng et al. signature [17].

The above risk factors were further extracted for construction of a combined signature using the LASSO COX model [19, 20]. As a result, we defined the combined signature as follows: CS = (0.008974621 × Age) + (1.617859481 × Grade) + (0.940077644 × Signature_1) + (0.006408624 × Signature_3). Here, Grade = 1 for lower grade glioma, and Grade = 2 for high grade glioma.

### Robustness test

We tested the robustness of the macrophage-related gene signature obtained using the multicellular network perturbation analysis in comparison with LASSO Cox signature and correlation network-based signature using a bootstrapping approach. We generated 100 random datasets by randomly sampling 50% of the samples from the TCGA datasets (i.e., validation set). The AUC values of ROC with respect to overall survival, 3-year survival and 5-year survival were computed. Wilcoxon rank sum test (one-tailed) *p* values were computed to assess the significance of the difference between the probability distributions of AUC values of competing signature.

## Results

### Construction and analysis of multicellular gene networks

The heatmaps of DEGs in TAMs (Additional file 1: Fig S1A) and TCs (Additional file 1: Fig S1B) across all samples demonstrated that the gene expression profile of the Reb samples was significantly different from that of Ep samples. The constructed sensitive network and sample-specific perturbation networks along with their topological attributes are shown in Additional file 1: Fig S2 and Fig S3–S6, respectively. A significant difference in the number of nodes and edges was observed between the sensitive and perturbation networks (Additional file 1: Fig S7). Other network topological attributes, including the network diameter, network centralization, and average number of neighbors, of the perturbation networks (Additional file 1: Fig S3–S6) were mainly higher than those of the sensitive network (Additional file 1: Fig S2). These results suggest that the perturbation networks had more complexity and that network rewiring might emerge during the acquisition and development of resistance to CSF1R inhibition.

**Fig. 2:**
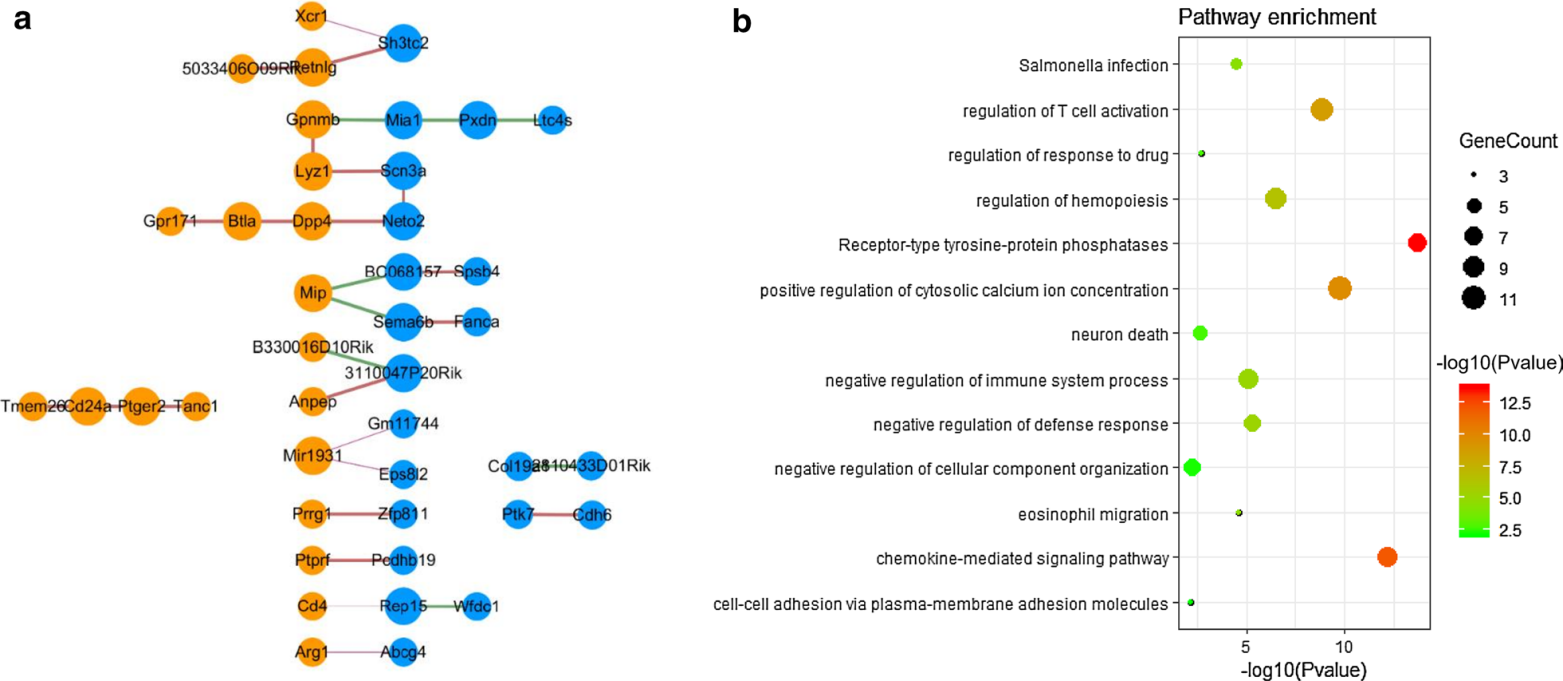
Differential network between drug-sensitive and drug-resistant multicellular gene networks and functional enrichment analysis. **a** A robust differential gene association network underlying glioma resistance to CSF1R inhibition. The node label represents the gene name. The size of the node represents its connectivity. The orange and blue nodes represent the genes in TAMs and TCs, respectively. **b** Pathway enrichment of the genes in the differential network

A robust differential network (Fig. 2a) containing 42 nodes and 30 edges was thus constructed. The genes in the differential network are listed in Additional file 1: Table S3. Interestingly, all edges in the differential network were correlation-gained edges, indicating that the addition of Reb samples improved the correlation coefficients, which is in accordance with the above results (Additional file 1: Fig S2–S7).

Functional enrichment analysis of genes in the differential network revealed that some pathways, including the chemokine-mediated signaling and receptor-type tyrosine-protein phosphatase pathways as well as the cell-cell adhesion via plasma membrane adhesion molecule pathway, were significantly enriched during the acquired resistance to CSF1R inhibition treatment (Fig. 2b). This result suggests that cell–cell interactions between TCs and TAMs might contribute to the resistance of glioma to CSF1R inhibition, which was supported by the previous experimental results [10, 23–25].

### Identification of the macrophage-related gene signature

**Fig. 3:**
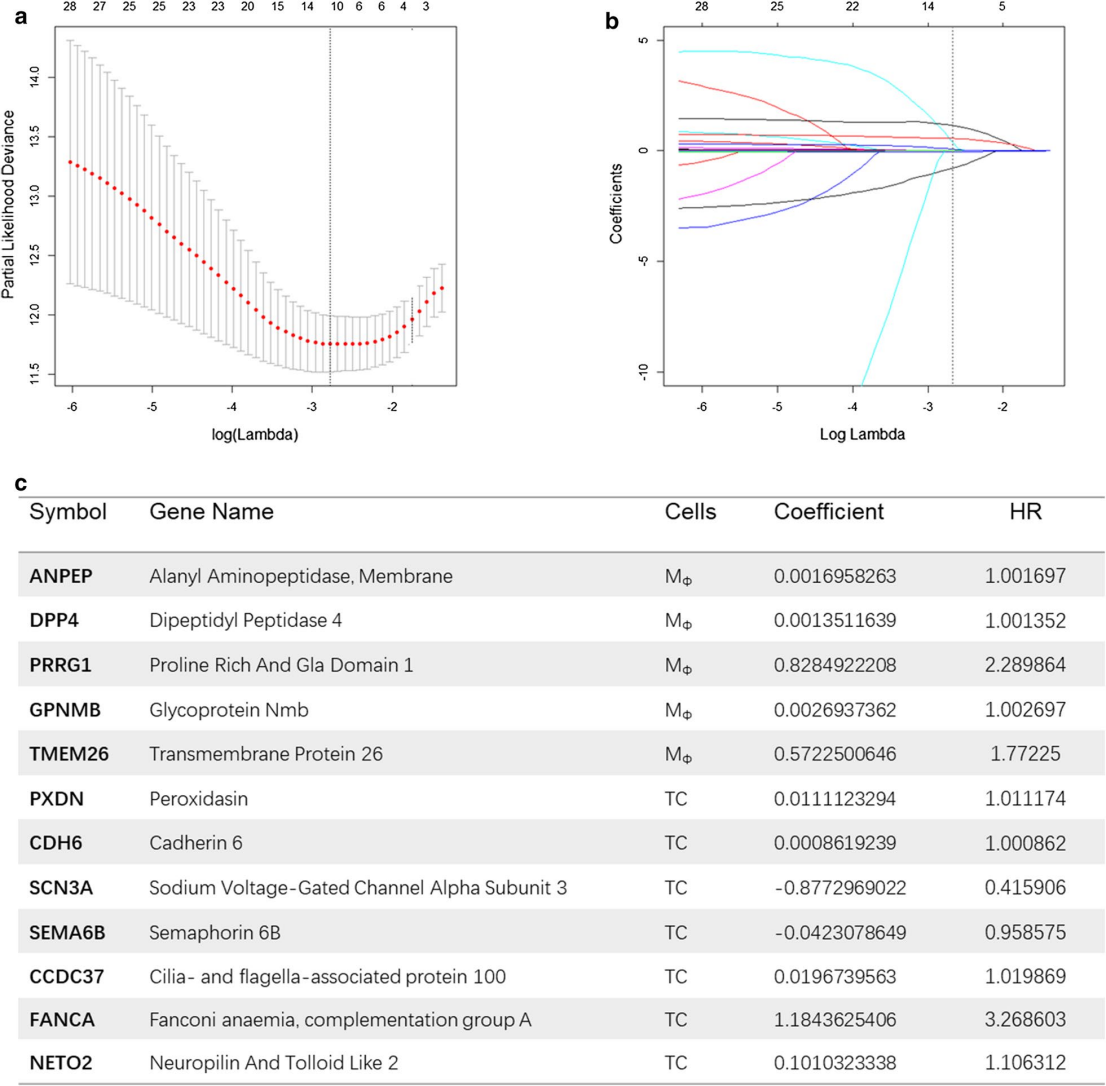
Macrophage-related gene signature identification from the differential network using LASSO regression. **a** tenfold cross-validation for tuning the parameter selection in the LASSO regression. The solid vertical lines represent the partial likelihood deviance with standard error. The dotted vertical lines denote the optimal values of the tuning parameter (λ) by minimum criteria, i.e., λ = 0.06226413 with ln(λ) = − 2.77637. **b** LASSO coefficient profiles of the 29 genes in the differential network. Each curve corresponds to evolution of the coefficient of each gene with respect to the ln(λ) during the LASSO regression. The dotted vertical lines denote the selected variables under the optimal tuning parameter. **c** The 12 genes selected by LASSO regression. The table lists each gene’s symbol, description, cell type (i.e., macrophages (M_Ф_) or tumor cells (TCs)) in the differential network, coefficient in the COX PH model and corresponding hazard ratio (HR)

We selected prognostic genes from the differential network using the LASSO regression method (see “Methods” section and Text S1). Figure 3a shows the selection of optimal tuning parameter (λ) of LASSO regression based on tenfold cross-validation of the learning set. Figure 3b shows the LASSO coefficient profiles of the 29 genes in the differential network. Each curve corresponds to evolution of the coefficient of each gene with respect to the change of the tuning parameters during the LASSO regression. Figure 3c lists the 12 genes selected under the optimal tuning parameter of LASSO. Five genes were located in macrophages (MФ) (i.e., ANPEP, DPP4, PRRG1, GPNMB, TMEM26), and 7 genes were located in TCs (i.e., PXDN, CDH6, SCN3A, SEMA6B, CCDC37, FANCA, NETO2). The coefficients of each gene in the COX PH model and the corresponding hazard ratios (HRs) were also listed and used to define a macrophage-related gene signature for predicting the prognosis of glioma patients. Accordingly, we formulated the following RS for each patient based on the expression levels of the selected genes: RS = (0.001695826 × ANPEP) + (0.001351164 × DPP4) + (0.828492221 × PRRG1) + (0.002693736 × GPNMB) + (0.572250065 × TMEM26) + (0.011112329 × PXDN) + (0.000861924 × CDH6) − (0.877296902 × SCN3A) − (0.042307865 × SEMA6B) + (0.019673956 × CCDC37) + (1.184362541 × FANCA) + (0.101032334 × NETO2). Furthermore, the univariate COX regression analysis (Additional file 1: Table S4) demonstrated that each signature gene was significantly associated with the survival of glioma patients.

### Validation of prognostic significance and accuracy of the macrophage-related gene signature

**Fig. 4:**
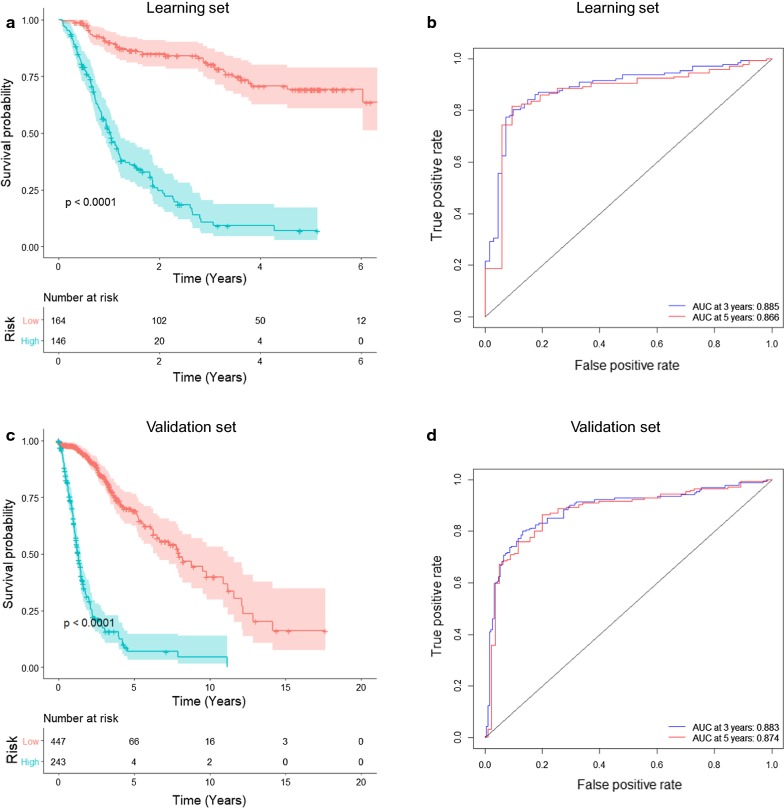
Prognostic significance of the macrophage-related gene signature in the learning and validation sets. **a** K–M survival analysis of glioma patients in the learning set. A risk score was formulated from the COX PH model based on the gene expression levels of the 12 selected genes. The patients were divided into the high-risk group (blue) and low-risk group (red). The statistical significance of the difference between two K–M survival curves was assessed using the log-rank test, with *p* values less than 0.0001. **b** Prognostic accuracy of the signature evaluated by the AUCs of the time-dependent ROCs with respect to the 3- and 5-year survival rates of glioma patients in the learning set. **c** Prognostic significance of the macrophage-related gene signature validated by the independent validation set. *p* values were less than 0.0001, as assessed by the log-rank test. **d** Prognostic accuracy of the macrophage-related gene signature validated by the AUCs of the time-dependent ROCs with respect to the 3- and 5-year survival rates of glioma patients in the independent validation set

We then investigated the prognostic significance and accuracy of the macrophage-related gene signature in both the learning and independent validation sets. Figure 4a shows the K–M curves for high-risk (blue) and low-risk (red) glioma patients in the learning set; significant differences were assessed with the log-rank test (*p* value less than 0.0001). The high-risk group of glioma patients tended to have a shorter survival time than the low-risk group. Figure 4b shows the prognostic accuracy of the risk signature evaluated by the AUCs of the time-dependent ROCs with respect to the 3- and 5-year survival rates of glioma patients in the learning set (AUC at 3 year: 0.92; AUC at 5 years: 0.891).

Moreover, we tested the prognostic significance of the macrophage-related gene signature using the independent validation set (Fig. 4c). The K–M curves demonstrated a significant difference between the survival rates of high- and low-risk patients (log-rank test *p* value less than 0.0001). Figure 4d shows the prognostic accuracy of the macrophage-related gene signature validated by the AUCs of the time-dependent ROCs with respect to 3- and 5-year survival rates of glioma patients in the validation set (AUC at 3 years: 0.818; AUC at 5 years: 0.836). These results demonstrated good prognostic value of the macrophage-related gene signature in glioma patients.

**Fig. 5:**
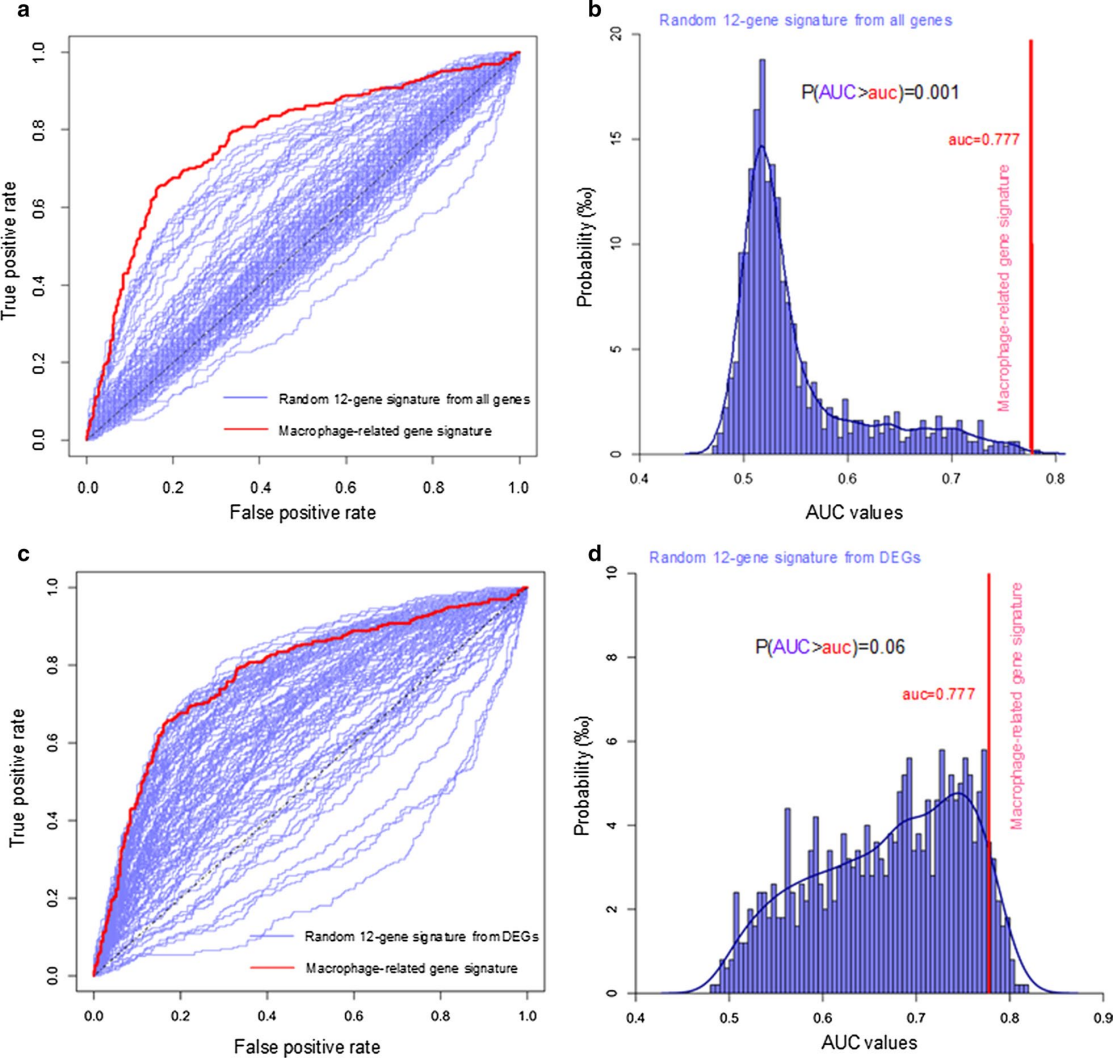
Evaluating statistical significance of prognostic accuracy in overall survival of the macrophage-related gene signature and competitors using a bootstrapping approach. **a**, **b** We randomly selected 12-gene sets from the whole transcriptome for 1000 times and compared their prognostic accuracy with that of the macrophage-related gene signature. **a** ROC curves of bootstrapped 12-gene signatures (blue, only 100 curves were shown) and macrophage-related gene signature (red), with respect to the overall survival of glioma patients in the validation set. **b** Probability distributions of the AUC values of the ROCs of random 12-gene signatures. The AUC of macrophage-related gene signature (red line, auc = 0.777) was shown for comparison. Probability P(AUC > 0.777) = 0.001. **c**, **d** We randomly selected 12-gene sets from the full list of DEGs for 1000 times and compared their prognostic accuracy with that of the macrophage-related gene signature. **c** ROC curves of bootstrapped 12-gene signatures (blue, only 100 curves were shown) and macrophage-related gene signature (red), with respect to the overall survival of glioma patients in the validation set. **d** Probability distributions of the AUC values of the ROCs of random 12-gene signatures. The AUC of macrophage-related gene signature (red line, auc = 0.777) was shown for comparison. Probability P(AUC > 0.777) = 0.06

Furthermore, we tested the significance of prognostic accuracy of the macrophage-related gene signature evaluated using a bootstrapping approach. We randomly selected 12-gene sets from the whole transcriptome for 1000 times and compared their prognostic accuracy with that of the macrophage-related gene signature. CGGA dataset was used for training and TCGA dataset for validation. Figure 5a shows ROC curve of the macrophage-related gene signature (red) against ROC curves of bootstrapped 12-gene signatures (blue, only 100 curves were randomly shown). Figure 5b shows the probability distributions of the AUC values of 1000 sets of random 12-gene signatures. The *p* value (0.001) from the permutation test justified the statistical significance of the prognostic accuracy of the macrophage-related gene signature.

**Fig. 6:**
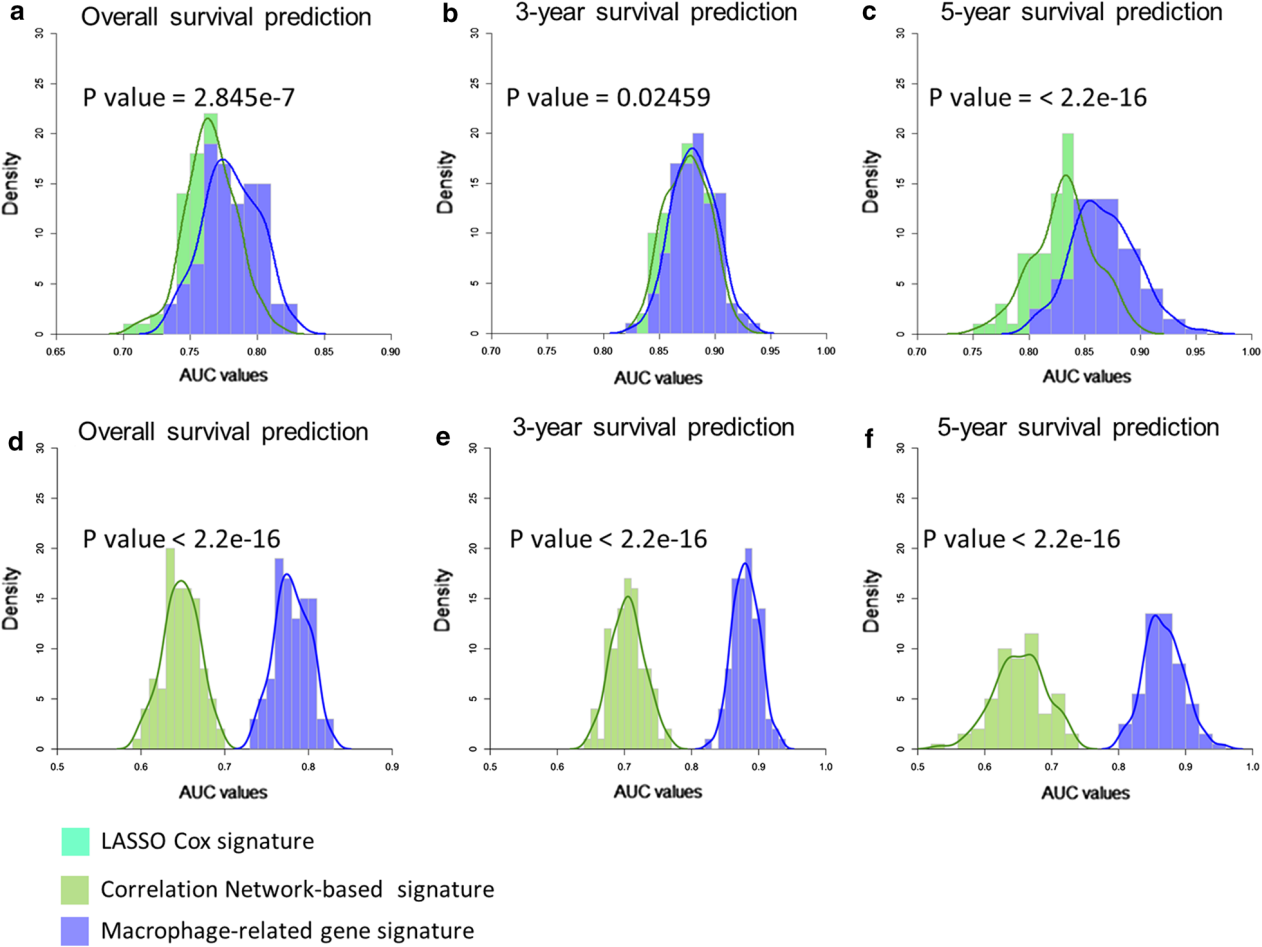
Robustness tests of the macrophage-related gene signature compared with signatures identified by LASSO Cox regression model (**a**–**c**) and correlation-network-based method (**d**–**e**). We generated 100 random datasets by randomly taking 60% of the samples from the validation set. The AUC values of ROC with respect to overall survival (**a**, **d**), 3-year survival (**b**, **e**) and 5-year survival (**c**, **f**) were computed. Wilcoxon rank sum test (one-tailed) *p* values were computed to assess the significance of the difference between the probability distributions of AUC values of the macrophage-related gene signature and the LASSO Cox signature or correlation-network-based signature. The macrophage-related gene signature showed good robustness and better accuracy than the LASSO Cox signature or correlation-network-based signature

For comparison, we also generated gene signatures using LASSO Cox regression model and correlation network-based method (see Method section). Additional file 1: Fig S8 shows the prognostic accuracies of LASSO Cox signature and correlation network-based signature with respect to 3-year and 5-year survival prediction. Although these two signatures performed well on the learning set, their predictive accuracies on the test sets were less than the macrophage-related gene signature from the network perturbation analysis. We further employed a bootstrapping approach to test and compare the robustness of these gene signatures derived from different methods. Figure 6 shows the AUC values of ROCs of these signatures with respect to overall survival, 3-year survival and 5-year survival. The macrophage-related gene signature exhibited better accuracy and robustness than the LASSO Cox signature and the correlation-network-based signature.

### The macrophage-related gene signature is predictive of the responses of gliomas to targeted therapeutics

**Fig. 7:**
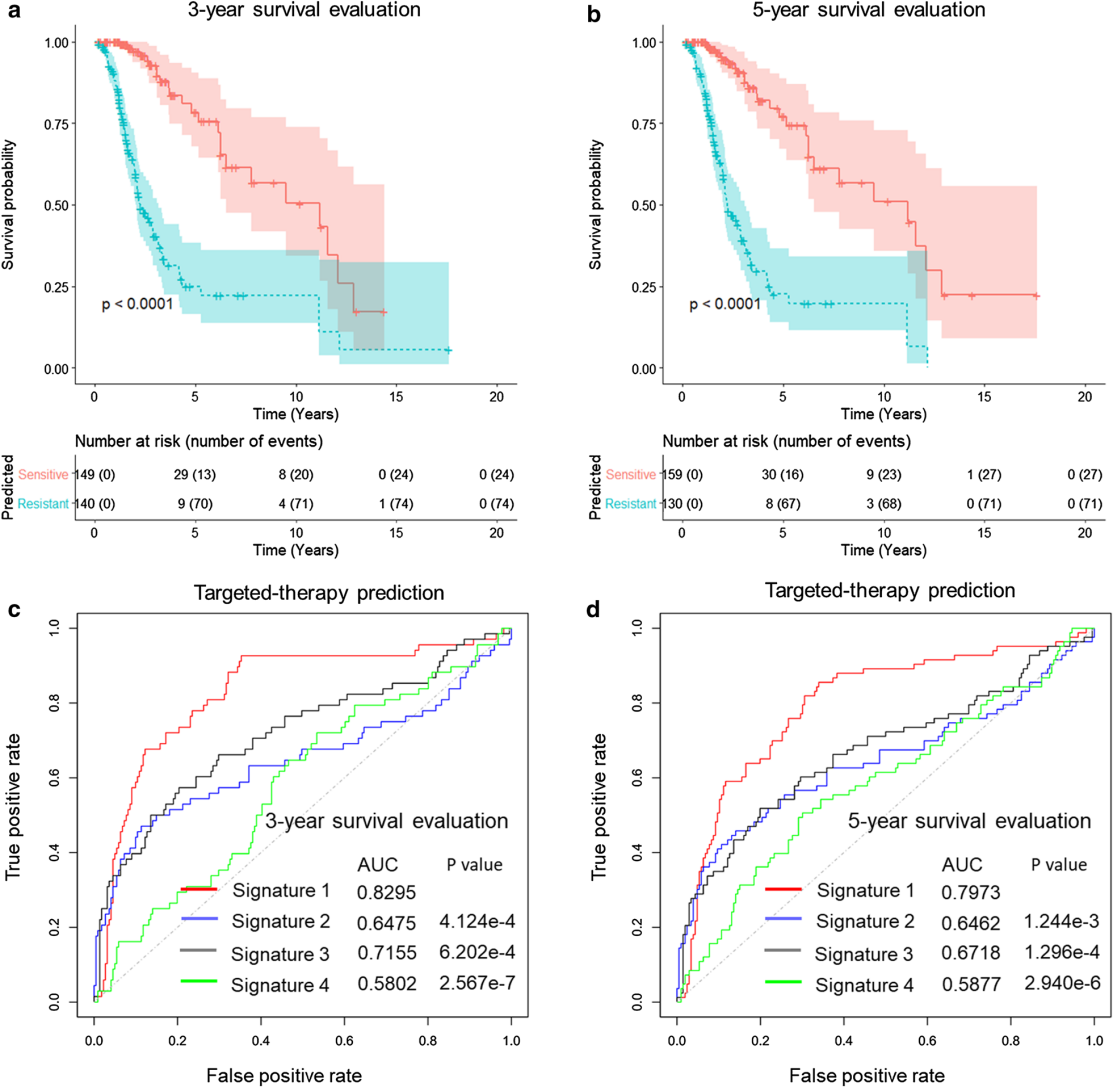
The macrophage-related gene signature was predictive of drug sensitivity/resistance to the targeted therapy. **a**, **b** Patients who received targeted therapy in the independent validation set were predicted to be drug-sensitive or drug-resistant based on the risk score calculated from the macrophage-related gene signature using optimal cutoff values according to the 3-year survival (**a**) or 5-year survival (**b)** outcomes after the molecularly targeted therapy. K–M survival curves of the patients predicted to be sensitive (blue) and resistant (red) were plotted, and the statistical significance was assessed using the log-rank test. All *p* values were less than 0.0001. **c**, **d** Accuracy of the macrophage-related gene signature for predicting drug sensitivity to the targeted therapy in glioma patients in comparison with other signatures. Signature 1: macrophage-related gene signature. Signature 2: EGFR gene signature. Signature 3: Cheng et al. signature. Signature 4: IGF1/IGF1R-mediated pathways signature. The prediction accuracies of these signatures evaluated by the 3-year survival (**c**) or 5-year survival (**d**) outcomes were assessed by the AUCs of the ROC curves based on the validation set. AUC values of each signature were shown, and *p* values were computed to assess the statistical significance of superior predictive power of the signature 1 against the other two signatures

Considering the important roles of macrophages in glioma progression and drug resistance [10–12, 26], a subset of TCGA patients who received targeted therapy were used to test the predictive power of the above macrophage-related gene signature for predicting drug sensitivity or resistance in glioma patients. The drug-resistance status of these patients was latent, we thus used the associated survival profiles as surrogate. The 3- or 5-year survival status (alive or dead) of each patient after the targeted therapy was used to evaluate the treatment outcome of the molecularly targeted therapeutics (sensitive or resistant). Each patient was predicted to be drug-sensitive (i.e., low-risk) or drug-resistant (i.e., high-risk) according to the optimal cutoff value of the RS using the time-dependent ROC method evaluated at 3-year or 5-year. Figure 7a, b shows the K–M survival curves of the predicted drug-sensitive patients (blue) and drug-resistant patients (red) as evaluated by 3-year survival ROC (Fig. 7a) or 5-year survival ROC (Fig. 7b), verifying distinct survival profiles between the two predicted groups of patients (log-rank test *p* values less than 0.0001).

To further assess the accuracy of different gene signatures for predicting the sensitivity or resistance to the targeted therapies, we compared 3 risk signatures that were designed for glioma patients: signature 1—the macrophage-related gene signature newly proposed in this study; signature 2—a conventional EGFR gene signature studied by many groups [15, 16]; signature 3—an immune-related gene signature, i.e., the Cheng et al. signature (FOXO3, IL6, IL10, ZBTB16, CCL18, AIMP1, FCGR2B, and MMP9) [17]; Signature 4—a gene signature based on IGF1/IGF1R-mediated pathways between macrophages and glioma cells (Quail et al. [10]), including several gene families in these pathways (PIK3R1, PIK3R2, PIK3R3, PIK3R4, PIK3R5, PIK3R6, PIK3AP1, AKT1, AKT2, AKT3, IGF1, IGF1R, IL4, IL4R, NFATC1, NFATC2, NFATC3, NFATC4, NFAT5, STAT6, CSF1 and CSF1R). We calculated the AUCs of the ROCs of these signatures to quantitatively assess and compare the accuracies of different gene signatures. Figure 7c shows the AUCs of the ROCs of these signatures for predicting drug sensitivity as evaluated by the 3-year survival outcomes (AUC of signature 1: 0.8295; AUC of signature 2: 0.6475; AUC of signature 3: 0.7155; AUC of signature 4: 0.5802). Figure 7d shows the AUCs of the ROCs of these signatures for predicting drug sensitivity as evaluated by the 5-year survival outcomes (AUC of signature 1: 0.7973; AUC of signature 2: 0.6462; AUC of signature 3: 0.6718; AUC of signature 4: 0.5877). Small *p* values computed using a bootstrap method [27] further demonstrated a superior predictive power of the macrophage-related gene signature compared with that of other signatures.

### The macrophage-related gene signature is an independent prognostic signature

The univariate and multivariate COX regression analyses for both the learning and validation datasets revealed that the macrophage-related gene signature retained prognostic significance for glioma patients when adjusted for both clinicopathologic risk factors (age, gender, grade) and other existing gene signatures (EGFR gene signature, Cheng et al. signature and IGF1/IGF1R pathways signature) (Table 1). These results indicated that the macrophage-related gene signature was independently correlated with the overall and 5-year survival of glioma patients.

**Table 1:** Multivariate COX regression analysis of clinicopathologic factors and four gene signatures for predicting overall survival and 5-year survival in the validation set

Variable	Univariate COX		Multivariate COX	
	*p* value	HR (95% CI)	*p* value	HR (95% CI)
*Overall survival*				
Age				
≥ 60 versus < 60	< 2e−16	4.8152 (3.687–6.288)	3.65e−5	1.900 (1.410–2.577)
Gender				
Male versus Female	0.111	1.2243 (0.954–1.571)	0.7084	1.051 (0.808–1.368)
Grade				
High- versus low-grade	< 2e−16	9.4994 (7.212–12.51)	2.05e−5	2.583 (1.6691–3.997)
Signature 1				
High- versus low-risk	< 2e−16	1.322 (1.276–1.369)	1.74e−10	1.191 (1.129–1.257)
Signature 2				
High- versus low-risk	6.11e−6	2.718 (1.762–4.193)	0.2901	1.223 (0.842–1.777)
Signature 3				
High- versus low-risk	< 2e−16	1.559 (1.469–1.655)	0.0196	1.124 (1.019–1.241)
Signature 4				
High- versus low-risk	0.00258	1.022 (1.008–1.036)	0.2511	1.008 (0.994–1.022)
*5*-*year survival*				
Age				
≥ 60 versus < 60	< 2e−16	4.887 (3.737–6.392)	6.56e−5	1.868 (1.374–2.539)
Gender				
Male versus Female	0.07	1.279 (0.980–1.669)	0.7605	1.044 (0.790–1.381)
Grade				
High- versus low-grade	< 2e−16	9.499 (7.212–12.51)	8.27e−5	2.418 (1.558–3.752)
Signature 1				
High- versus low-risk	< 2e−16	1.380 (1.329–1.432)	1.46e−12	1.234 (1.164–1.308)
Signature 2				
High- versus low-risk	8e−06	2.775 (1.779–4.328)	0.4696	1.149 (0.789−1.672)
Signature 3				
High- versus low-risk	< 2e−16	1.581 (1.488–1.679)	0.0283	1.121 (1.012–1.242)
Signature 4				
High- versus low-risk	0.00278	1.023 (1.008–1.038)	0.5823	1.004 (0.9901–1.018)

Signature 1: macrophage-related gene signature. Signature 2: EGFR gene signature. Signature 3: Cheng et al. signature. Signature 4: IGF1/IGF1R pathways signature. The macrophage-related gene signature retained prognostic significance for both the overall survival and 5-year survival of glioma patients, indicating that the macrophage-related gene signature is an independent risk factor for glioma patients

**Fig. 8:**
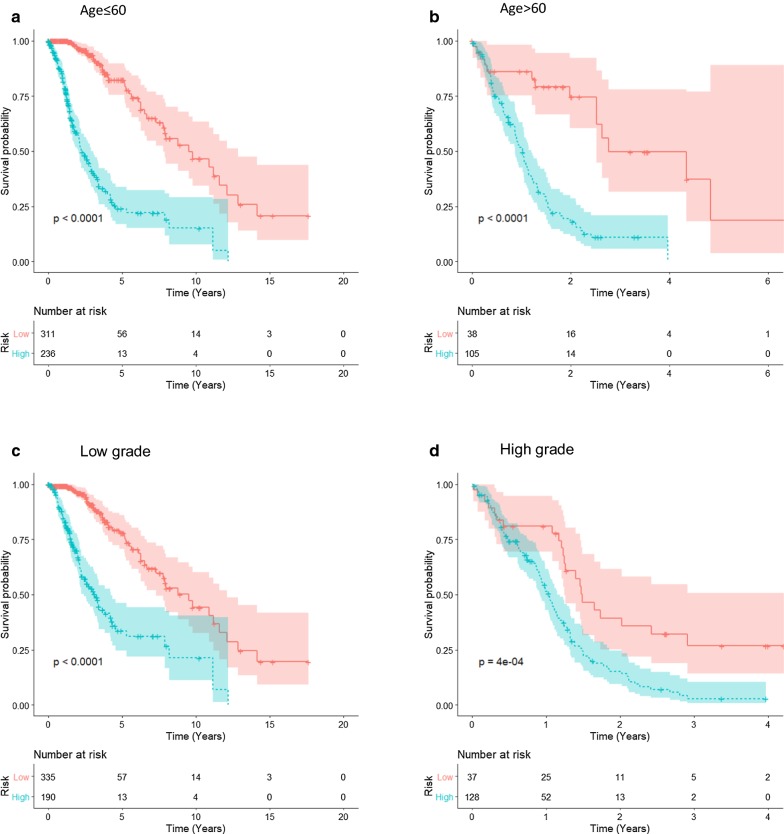

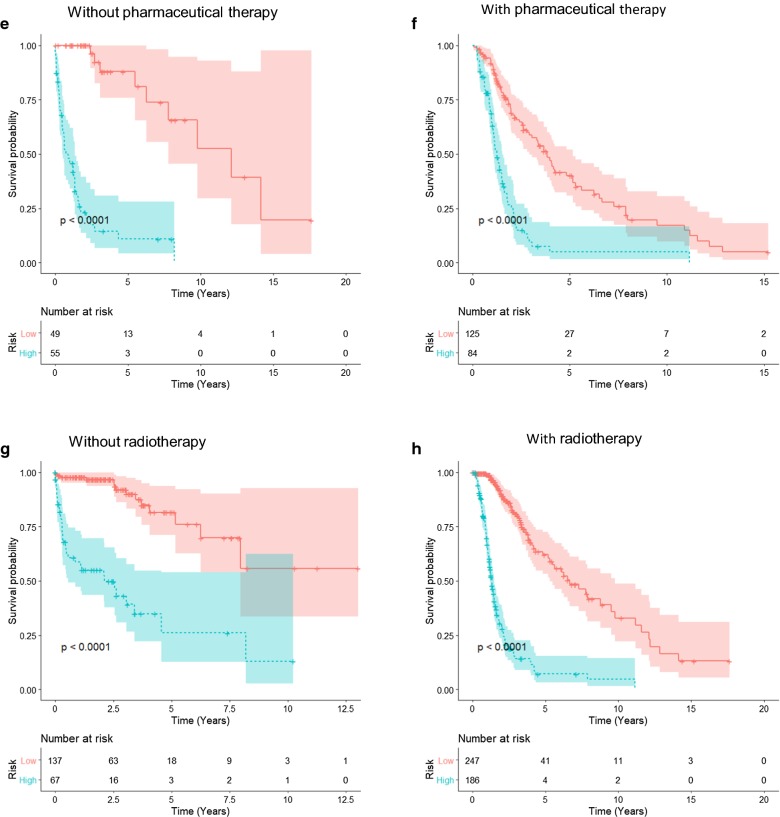
Prognostic significance of the macrophage-related gene signature in the stratified cohorts. **a**–**d** Prognostic significance of the macrophage-related gene signature in different cohorts stratified by age (age ≤ 60 or age > 60, panels A and B, respectively) or grade (low grade and high grade, panels C and D, respectively). **e**–**h** The macrophage-related gene signature retained prognostic significance for glioma patients treated with or without pharmaceutical therapy (**e** and **f**) and radiotherapy (**g** and **h**). Optimal cutoff values were used to determine high- and low-risk groups in each stratified cohort, and the statistical significance of the difference between two K–M survival curves was assessed using the log-rank test

To further explore the prognostic value of the macrophage-related gene signature in stratified cohorts, patients were first classified by 2 important clinicopathological factors, age and grade, that significantly correlated with the prognosis of glioma patients (Table 1). Figure 8a–d shows the prognostic significance of the macrophage-related gene signature in different glioma cohorts stratified by age (Fig. 8a, b) or grade (Fig. 8c, d). In all of these cohorts, patients were classified as high- versus low-risk groups using cut point from ROCs, and the high-risk patients had a significantly shorter overall survival than low-risk patients. Subsequently, patients who received pharmaceutical therapy and radiotherapy were utilized to validate the prognostic significance of the macrophage-related gene signature. Figure 8e–h demonstrates that the macrophage-related gene signature retained prognostic significance for glioma patients treated with or without pharmaceutical therapy (Fig. 8e, f) and radiotherapy (Fig. 8g, h). These results indicated that the macrophage-related gene signature could accurately identify patients with an unfavorable prognosis regardless of their clinicopathological and treatment characteristics.

**Fig. 9:**
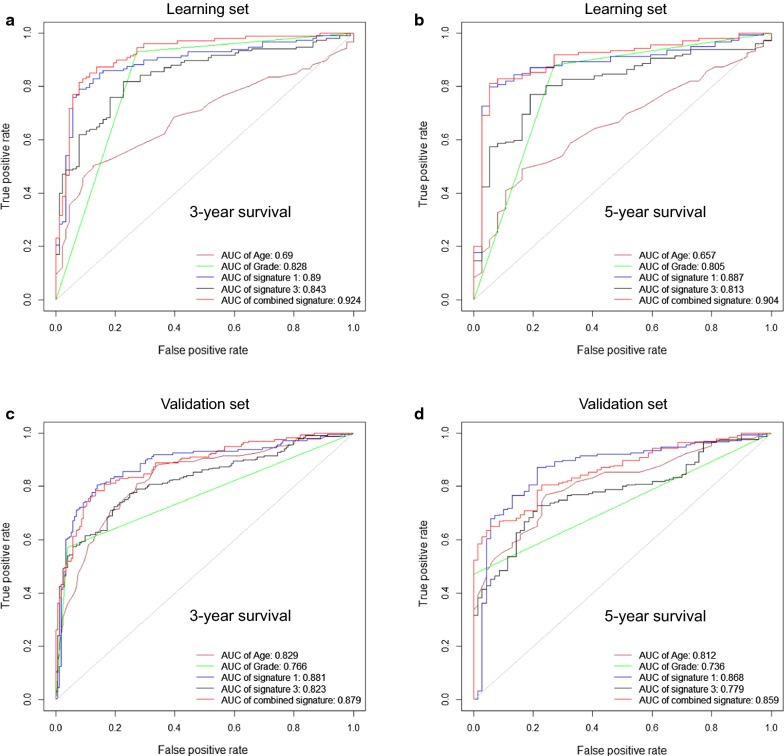
Time-dependent ROC curves comparing the prognostic accuracy of the macrophage-related gene signature with clinicopathological risk factors and other existing gene signatures or their combination. **a**, **b** Comparisons of the prognostic accuracy by age, grade (low grade or high grade), signature 1 (i.e., macrophage-related gene signature), signature 3 (i.e., Cheng et al. signature) and the combined signature using the learning set with respect to 3-year survival (**a**) or 5-year survival (**b**). **c**, **d** Comparisons of the prognostic accuracies with respect to 3-year survival (**c**) or 5-year survival (**d**) using the validation set

We further examined whether combining the macrophage-related gene signature with clinicopathological risk factors and other existing gene signatures could significantly improve the prognostic accuracy of the risk signature. The time-dependent ROC curves (Fig. 9) compared the prognostic accuracy by age, grade, signature 1 (i.e., macrophage-related gene signature), signature 3 (i.e., Cheng et al. signature) and the combined signature (see the definition in the Methods section). The AUCs of ROC curves in both the learning dataset (Fig. 9a, b) and the validation dataset (Fig. 9c, d) showed that the combined signature outperformed other risk signatures except the macrophage-related gene signature for predicting the 3- and 5-year survival rates of glioma patients. Noticeably, the AUC of the ROC of the macrophage-related gene signature was rather close to that of the combined signature in the learning dataset (Fig. 9a, b), or even higher in the validation dataset (Fig. 9c, d). These results indicated that the macrophage-related gene signature is an independent prognostic signature and possessed convincingly strong and robust prognostic power in comparison to the clinicopathological factors and other existing risk signatures.

## Discussion

Several studies have reported that the tumor microenvironment plays important roles in glioma progression and therapeutic response [10–12, 28–31]. TAMs, a type of immune cell, account for the majority of nonneoplastic cells in the glioma microenvironment [26]. However, limited studies have investigated macrophage-based molecular biomarkers for glioma prognostication and therapeutic response prediction. In this study, we first identified and validated a macrophage-related gene signature that has a strong prognostic significance and accuracy for glioma patients.

The conventional node biomarker method identifies biomarkers using a set of single genes or molecules and does not consider interactions between these genes [32–38]. In recent years, a network biomarker method has been proposed to identify biomarkers of cancers or drug resistance using a network-based analysis approach, which takes into account gene interactions and uses aggregates of genes/proteins in networks for prediction [39–41]. However, these previous network-based approaches mainly focused on intracellular gene networks within TCs and did not explicitly or systematically consider intercellular interactions, particularly tumor–microenvironment interactions. In this study, we built multicellular gene networks connecting TAMs and TCs to identify gene signatures for predicting prognosis and drug resistance in glioma patients, which provided systems biology insights into tumor cell–microenvironment interactions in cancer progression and therapeutic resistance. Furthermore, a robust differential gene network was constructed using the network perturbation analysis method, which is suitable for small sample sizes. The resulting differential gene pairs (Fig. 2a) not only reflected the intracellular gene coexpression patterns within TCs and TAMs but also revealed the intercellularly correlated genes between these cell types during the acquisition of drug resistance.

We compared the macrophage-related gene signature with other existing signatures, and our signature showed better accuracy for predicting the survival outcomes of glioma patients who received molecularly targeted therapies (Fig. 7). We speculate that the superiority of our signature lies in the fact that it considered the interaction between TCs and the microenvironment during tumor progression. Although we considered only TAMs, which constitute a fraction of microenvironmental cell types, the identified TAM-TC gene signature outperformed the existing signatures that focused on TCs or immune genes only, which is an intriguing finding for investigating the prognostic significance of microenvironment-mediated gene signatures. The multicellular gene network approach developed herein provides a promising strategy to systematically integrate genes in both TCs and microenvironmental cells and thus identify more robust and accurate signatures or biomarkers.

**Fig. 10:**
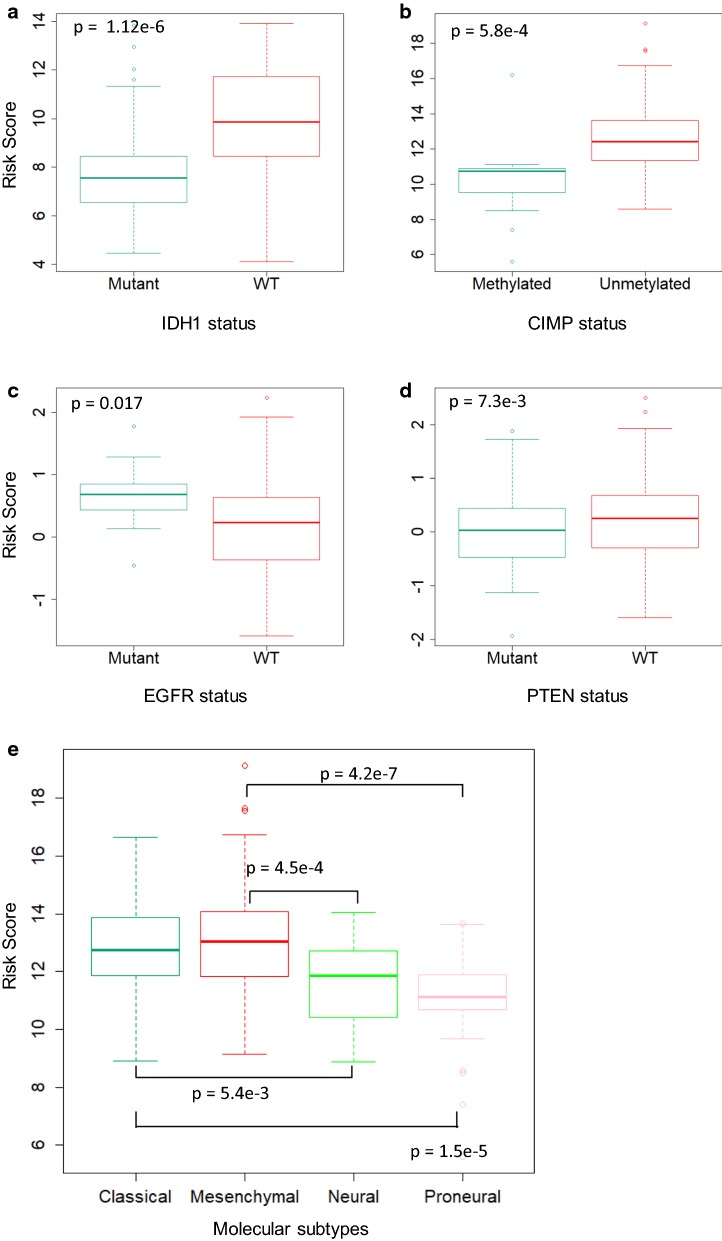
Distribution of the macrophage-related gene signature in patients stratified by IDH1 mutation status (**a**), CIMP status (**b**), EGFR status (**c**), PTEN status (**d**) and molecular subtypes (**e**). Wilcoxon rank sum test (two sided) *p* value was used to assess the statistical significance of the difference between each of the two comparison groups

In addition, we assessed the association between the macrophage-related gene signature and frequent genetic and genomic alterations in gliomas by exploring other genomic characteristics that are available within the TCGA or CGGA databases. Figure 10 shows the distribution of the risk scores evaluated by macrophage-related gene signature in patients from the TCGA set stratified by IDH1 mutation status (Fig. 10a) or methylator CIMP status (Fig. 10b), and in patients stratified by EGFR mutation status (Fig. 10c) or PTEN mutation status (Fig. 10d) with the information available from the CGGA set. The signature value was different between cases stratified by IDH1 mutation status, CIMP status, EGFR mutation status and PTEN status. We also examined the distribution of the risk scores in patients stratified by molecular subtypes with the information available from the TCGA set (Fig. 10e). Significant differences of risk values were observed between several pairs of different subtypes (classical vs. neural, classical vs. proneural, mesenchymal vs. neural, and mesenchymal vs. proneural). These results suggested the associations of the macrophage-related gene signature with genetic and genomic alterations in gliomas and molecular subtypes. In future studies, we will develop an ensemble model that integrates multiple gene signatures designed for different subgroups of glioma patients stratified by molecular subtypes or major genomic mutations in IDH1, CIMP, EGFR and PTEN in glioma cohorts to further improve the predictive accuracy.

The established macrophage-related gene signature includes both protective genes (SCN3A, SEMA6B) and risk-increasing genes (PXDN, CDH6, ANPEP, CCDC37, DPP4, GPNMB, FANCA, NETO2, PRRG1, TMEM26) for predicting the prognosis of glioma patients (Fig. 3). This signature could be regarded as a macrophage-related protective and risky pattern of TC–TAM interactions in gliomas, which is consistent with the balance between the antitumorigenic M1 phenotype and protumorigenic M2 phenotype of TAMs in gliomas.

Additional file 1: Table S5 lists the experimental evidences for functional roles of the five macrophage-related genes in cancer progression and/or drug resistance. For instance, ANPEP was reportedly involved in cell migration and tumor metastasis [42–48]. ANPEP and TMEM26 were associated with drug response [49, 50]. Remarkably, the important role of DPP4 in anti-tumor immunity [51] has been revealed in many cancers including glioblastomas [52]. The inhibition of DPP4 was found to improve both naturally occurring tumor immunity and immunotherapy by enhancing lymphocyte trafficking [53]. Our results may therefore implicate new macrophage-targeting treatment strategies to improve clinical outcomes. The genes in the macrophage-related gene signature could be employed as stand-alone targets or in combination with the existing targeted therapies, attributing to their prognostic significance and association with drug resistance. For example, GPNMB has been found to be highly upregulated in human glioma-associated microglia/macrophages, which are the predominant source of GPNMB transcripts [54]. High GPNMB expression was found to be associated with poor prognosis in human glioblastoma. Furthermore, GPNMB has also been indicated as a potential molecular therapeutic target in patients with glioblastoma [55].

The advantages of our study originate from the use of a multicellular network-based gene screening approach, large population databases for learning and validation and robust risk signature identification methods. In future studies, we will investigate the functions and mechanisms of the 12 genes alone and in combination to verify their clinical applicability. The capability of the signature identified herein for predicting drug resistance should be further validated by prospective studies in the future. We will also leverage single cell RNA-seq data to construct multilayer networks that connect tumor microenvironmental cells to tumor cells [56], which was anticipated to improve the biological interpretation and predictive accuracy of the biomarkers for predicting prognosis and therapeutic response.

## Conclusions

In summary, we developed a multicellular gene network approach to investigate the role of TC–TAM interactions in the progression and therapeutic responses of gliomas. The identified macrophage-related gene signature showed good accuracy for predicting the prognosis and targeted therapeutic responses of glioma patients. The multicellular gene network-based signature provided mechanistic insights into microenvironment-mediated drug resistance and implied that combining current targeted therapies with macrophage-targeted therapy might improve the long-term treatment outcomes of glioma patients.

## Additional files

**Additional file 1: Text S1.** Detailed description of the methods. **Table S1.** Scheme of sample grouping. **Table S3.** List of genes in the differential network. **Table S4.** Univariate Cox regression analysis for the association of each signature gene with the overall survival of glioma patients. **Table S5.** Experimental evidences for functional roles of the eight macrophage-related genes in cancer progression and/or drug resistance. **Figure S1.** Differential gene expression profiles of TAMs and TCs between the Endpoint and Rebound groups. **Figure S2.** TC-TAM sensitive network and its topological attributes. **Figure S3.** EpUReb1 sample-specific TC-TAM network and its topological attributes. **Figure S4.** EpUReb2 sample-specific TC-TAM network and its topological attributes. **Figure S5.** EpUReb3 sample-specific TC-TAM network and its topological attributes. **Figure S6.** EpUReb4 sample-specific TC-TAM network and its topological attributes. **Figure S7.** Network topological analysis of the sensitive network and 4 sample-specific resistant networks. **Figure S8.** Prognostic accuracies of LASSO Cox signature and correlation network-based signature. Supplementary references.

**Additional file 2: Table S2.** Supplementary tables for the details of patient sample information from CGGA and TCGA.

## Data Availability

The datasets including the RNA-seq data in mice (GSE69104), clinical information and the gene expression data of glioma patients analyzed during the current study are available in the GEO (https://www.ncbi.nlm.nih.gov/geo/query/acc.cgi?acc=GSE69104), TCGA database (https://cancergenome.nih.gov/) and CGGA database (http://www.cgga.org.cn/). The source R scripts for analysis in this study were available at https://github.com/dongbusun/MCGN-MS.

## Abbreviations

TME: tumor microenvironment
TC: tumor cells
TAMs: tumor-associated macrophages and microglia
DEG: differentially expressed gene
PCC: Pearson correlation coefficient
AUC: area under curve
ROC: receiver operating characteristic curve
