# Supplementary material for: Multicellular gene network analysis identifies a macrophage-related gene signature predictive of therapeutic response and prognosis of gliomas
Source: J Transl Med. 2019 May 16;17:159. doi: 10.1186/s12967-019-1908-1 (PMC6524242; doi:10.1186/s12967-019-1908-1)
Supplement: Supplementary file 1 — Additional file 1: Text S1. Detailed description of the methods. Table S1. Scheme of sample grouping. Table S3. List of genes in the differential network. Table S4. Univariate Cox regression analysis for the association of each signature gene with the overall survival of glioma patients. Table S5. Experimental evidences for functional roles of the eight macrophage-related genes in cancer progression and/or drug resistance. Figure S1. Differential gene expression profiles of TAMs and TCs between the Endpoint and Rebound groups. Figure S2. TC-TAM sensitive network and its topological attributes. Figure S3. EpUReb1 sample-specific TC-TAM network and its topological attributes. Figure S4. EpUReb2 sample-specific TC-TAM network and its topological attributes. Figure S5. EpUReb3 sample-specific TC-TAM network and its topological attributes. Figure S6. EpUReb4 sample-specific TC-TAM network and its topological attributes. Figure S7. Network topological analysis of the sensitive network and 4 sample-specific resistant networks. Figure S8. Prognostic accuracies of LASSO Cox signature and correlation network-based signature. Supplementary references. [file 12967_2019_1908_MOESM1_ESM.pdf]

Supplementary Information

Multicellular gene network analysis identifies a macrophage-related gene signature predictive of therapeutic response and prognosis of gliomas

Text S1. Detailed description of the methods.

Table S1. Scheme of sample grouping.

Table S3. List of genes in the differential network.

Table S4 Univariate Cox regression analysis for the association of each signature gene with the overall survival of glioma patients.

Table S5. Experimental evidences for functional roles of the eight macrophage-related genes in cancer progression and/or drug resistance.

Figure S1. Differential gene expression profiles of TAMs and TCs between the Endpoint and Rebound groups.

Figure S2. TC-TAM sensitive network and its topological attributes.

Figure S3. $EpUReb_1$ sample-specific TC-TAM network and its topological attributes.

Figure S4. $EpUReb_2$ sample-specific TC-TAM network and its topological attributes.

Figure S5. $EpUReb_3$ sample-specific TC-TAM network and its topological attributes.

Figure S6. $EpUReb_4$ sample-specific TC-TAM network and its topological attributes.

Figure S7. Network topological analysis of the sensitive network and 4 sample-specific perturbation networks.

Figure S8. Prognostic accuracies of LASSO Cox signature and correlation network-based signature.

Supplementary references

Text S1. Detailed description of the methods

RNA-seq data collection and analysis

The RNA-seq data of tumor cells (TCs) and tumor-associated macrophages (TAMs) in mice bearing gliomas were downloaded from the GEO website (https://www.ncbi.nlm.nih.gov/geo/query/acc.cgi?acc=GSE69104) with the accession number GSE69104 [1]. A CSF1R inhibitor, BLZ945, was used to treat the mice. The mice were divided into 3 groups, i.e., Vehicle (Veh), Endpoint (Ep, i.e., drug-sensitive), and Rebound (Reb, i.e., drug-resistant) tumors. Because our study was designed to construct a gene association network that connects TCs and TAMs, we selected TC-TAM paired samples that contained gene expression data for both TCs and TAMs but did not consider TC-TAM unpaired samples that contained only TCs or TAMs.

The collected dataset (*N*=15) included *S* (=6) samples of sensitive mice in the Ep group, *R* (=4) samples of mice in the Reb group, and *N*-*S*-*R* (=5) Veh samples. Therefore, the sample set $\mathrm{M} = (s_1, s_2, \ldots, s_N)$ could be divided into the following three subsets:

$$Ep = (s_1, s_2, \ldots, s_S). \tag{S1}$$

$$Reb = (s_{\mathrm{S+1}}, s_{\mathrm{S+2}}, \ldots, s_{\mathrm{S+R}}), \tag{S2}$$

$$Veh = (s_{\mathrm{S+R+1}}, s_{\mathrm{S+R+2}}, \ldots, s_N). \tag{S3}$$

Each sample contained the gene expression data of two cell types, i.e., TCs and TAMs. We denote

$$s_i = (c_{i,1}, c_{i,2}), (i = 1,2, \ldots, N), \tag{S5}$$

where $c_{i,1}$ and $c_{i,2}$ represent the TAMs and TCs of the i-th sample. The expression data of genes in TCs or TAMs could be described as follows:

$$C_{i,p} = \left(g_{i,p,1},\ g_{i,p,2}, \cdots, \text{[illegible]} \cdots \text{[illegible]}, \left(i = 1, \cdots \text{[illegible]} 1,2;\ r = 1, \cdots \right.\right. \tag{S6}$$

where $g_{i,p,r}$ represents the expression of gene *r* in cell type *p* (TC or TAM) of the i-th sample.

We normalized the gene expression levels of each gene in the Rebound (Reb) and Endpoint (Ep) samples with respect to their mean expression levels in the Vehicle (Veh) samples to evaluate the relative changes in drug response. For each gene r, we performed the following calculation:

$$\bar{v}_{p,r} = \frac{1}{N - (S + R)} \sum_{i=S+R+1}^{N} g_{i,p,r} \tag{S7}$$

$$g'_{i,p,r} = \log 2 \left(\frac{g_{i,p,r}}{\bar{v}_{p,r}} \right) \tag{S8}$$

where $g'_{i,p,r}$ represents the normalized expression level of gene r in cell type *p* (TC or TAM) of the i-th sample, and $\bar{v}_{p,r}$ denotes the mean expression of gene r in cell *p* of the Veh sample. There were *S* (=6) samples of Ep mice and *R* (=4) samples of Reb mice. *N* (=15) is the number of total samples, and thus, *N-(S+R)* is the number of Veh samples. Note that each sample included both two type cells (TC and TAM).

Selection of differentially expressed genes

We used a t-test and fold change to select the differentially expressed genes (DEGs) in TCs or TAMs between the Reb and Ep groups.

Let $x_i = \left(g'_{1,p,i}, g'_{2,p,i}, \cdots \right.$ [illegible] and $y_i = \left(g'_{S+1,p,i}, g'_{S+2,p,i}, \cdots \right.$ [illegible] $\left. \right)$ denote the normalized expression of each gene i in TCs (*p*=1) or TAMs (*p*=2) from the Reb and Ep samples, respectively. We calculated the two-sided t-test p-value for each gene in TAMs and TCs, which were adjusted by false discovery rate (FDR). When the FDR-adjusted p-value was less than 0.05, we considered it to be statistically significant. If the p-value of a gene in TAMs was less than 0.05, this gene was collected into a set called Q_1; if the p-value of a gene in TCs was less than 0.05, this gene was collected into a set called Q_2. We then calculated the fold change of the expression of each gene in sets Q_1 and Q_2 between the Reb and Ep samples as follows:

$$f_i = \left| \bar{x}_i / \bar{y}_i \right| . \quad \text{(S13)}$$

where $\bar{x}_i$ and $\bar{y}_i$ represent the mean value of expression levels of gene i in the Reb and Ep samples, respectively. According to the values of f_i, we selected the top 50 genes in each of Q_1 and Q_2, which constituted the DEG sets of TAMs and TCs, denoted as U_1 and U_2, respectively. The heatmaps of DEGs in TAMs (**Figure S1A**) and TCs (**Figure S1B**) across all samples demonstrated that the gene expression profile of the Reb samples was significantly different from that of Ep samples.

Sample-specific multicellular gene correlation network

We used the following Pearson's correlation coefficient (PCC) to measure the correlation between each pair of genes within U_1 (the DEG sets of TAMs) or U_2 (the DEG sets of TCs) and between U_1 and U_2.

$$PCC_n = \frac{\sum_{i=1}^{n} (x_i - \bar{x}) \cdot (y_i - \bar{y})}{\sqrt{\sum_{i=1}^{n} (x_i - \bar{x})^2} \cdot \sqrt{\sum_{i=1}^{n} (y_i - \bar{y})^2}} \quad \text{(S14)}$$

where $x=(x_1, x_2, \ldots, x_n)$ and $y=(y_1, y_2, \ldots, y_n)$ represent the expression levels of the two genes, and $\bar{x}$ and $\bar{y}$ represent their mean values, respectively. *n* is the sample size to be specified in the following text.

We first constructed the sensitive network (**Figure 1A**) based on the PCCs calculated from the sensitive samples (i.e., Ep samples, *n*=S). The nodes in the sensitive network were DEGs in U_1 and U_2. The edges in the sensitive network were determined by the PCCs (|PCC|>0.95 and p-value < 0.05) between each pair of nodes. The nodes were grouped into TC class and TAM class. Therefore, the sensitive TC-TAM gene network consisted two types of edges, i.e., 'intracellular edges' within TCs or TAMs and 'intercellular edges' between TCs and TAMs.

Given the small sample size of the Reb group, based on the work of one of our authors [2], we developed a network perturbation analysis method by combining each single Reb sample with all reference samples (all samples in the Ep group) for network construction and analysis. More specifically, we then generated 4 sets of perturbed samples by adding each single Reb sample (Reb_i, *i*=1, 2, 3, 4) to the reference samples (i.e., sensitive samples) and constructed 4 resistant sample-specific gene correlation networks. The edges with high PCCs (|PCC|>0.95 and p-value < 0.05) were utilized to build resistant TC-TAM gene correlation networks.

As a result, we constructed a sensitive network (i.e., reference network) and 4 perturbation networks. These networks are multicellular gene networks since they not only convey gene associations within TCs or TAMs but also build connections between these two types of cells (**Figure 1B**). The multicellular gene network approach developed in this study provides a way to dissect the potential interactions between tumor cells and the microenvironmental cells during the acquision and development of drug resistance.

Differential network

The addition of each single Reb sample induced differences between the reference and perturbation networks. If the gene expression profile in the added sample Reb_i was similar to that in the Ep samples, the perturbation of the PCC was negligible. However, if some gene expression levels were remarkably different between the single Reb sample and the Ep samples, significant changes in the PCCs of certain gene-pairs were induced when Reb_i was added to the reference samples.

For network perturbation analysis (**Figure 1C**), the robust significantly different PCCs (ΔPCC_i = $PCC_{S+i} - PCC_S$, *i*=1,2,3,4) of gene pairs were utilized to construct a differential network. Specifically, we selected significantly different edges, represented by ΔPCC, by setting a threshold of |ΔPCC|>0.5 for each perturbation network versus the sensitive network. If a gene-pair association was significantly changed in at least 3 perturbation networks compared to the reference network, this edge was selected as a robust differential edge, which constituted the final differential network.

For further network analysis, we defined three types of edges in the differential network: correlation-gained edges (ΔPCC>0), correlation-lost edges (ΔPCC<0), and correlation-invariant edges (ΔPCC=0). The patterns of such edges in the differential network might provide biological insights to better understand the acquisition of drug resistance.

We used R script to perform data analysis and network construction and used 'Cytoscape' software to visualize the constructed multicellular gene networks.

Differential network-based COX regression

The differential network captures the robust topological differences between the sensitive network and perturbation networks. Therefore, the differential network reflects the potential change in the gene association across TCs and TAMs during the acquisition and development of drug resistance. The genes in the differential network should play important roles in promoting tumor growth even under drug treatment conditions. We hypothesized that the expression levels of genes in the differential network are associated with the survival outcomes of glioma patients. Therefore, we next developed a differential network-based COX regression method to identify prognostic gene signatures for glioma patients.

We collected clinical information and RNA-seq gene expression data from glioma patients in the CGGA database (http://www.cgga.org.cn/) and TCGA database (https://cancergenome.nih.gov/). We also mapped the gene symbols in the initial mouse RNA-seq data (contains 23337 genes) to those of genes in homo sapiens, resulting in a list of 16761 mapped genes. The names of mouse genes in the differential network were thus mapped to 29 candidate genes in human. The names of genes in the differential network were thus mapped to 29 candidate genes, constituting the gene set H . By matching both the patient sample IDs and the gene names from the clinical information and gene expression data, a cohort of 310 glioma patients in the CGGA dataset and an independent cohort of 690 glioma patients in the TCGA dataset were prepared for learning and validation, respectively.

The available survival data are in the form $(y_1, \mathrm{X}^1, \delta_1)$, …, $(y_{\mathrm{N}}, \mathrm{X}^N, \delta_N)$. The survival time y_i is the observed event time if δ_i =1 and right censored if δ_i =0. X^i denotes the expression levels of genes in the differential network for the *i*-th patient, and N=690 and 310 for the TCGA and CGGA datasets, respectively. The COX proportional hazards (PH) model assumes that

$$H(t \mid X) = H_0(t) \cdot \exp\left(\sum_{i=1}^{m} \beta_i \cdot x_i\right), \qquad \text{(S16)}$$

where $H(t \mid X)$ is the hazard function at time *t* for each patient given predictor values $X = (x_1, x_2, \ldots, x_m)$, x_i is the expression level of gene *i* involved in the differential network, and *m* is the number of genes in gene set H . $H_0(t)$ is an arbitrary baseline hazard function. β_i is the regression coefficient of gene *i* in the COX PH model.

The likelihood of the above model is defined as follows:

$$L(\beta) = \prod_{r \in D} \frac{\exp\left(\beta^T X^{j_r}\right)}{\sum_{j \in R_r} \exp\left(\beta^T X^j\right)}, \qquad \text{(S17)}$$

where *D* is the set of indices of the failures, R_r is the set of indices of the individuals at risk at time t_r, and j_r is the index of the failure at time t_r.

We estimated the parameters $\beta=(\beta_1,\cdots$ in the above COX PH model using the following LASSO-type log-partial likelihood method:

$$\hat{\beta} = \arg\min_{\beta}(\log L(\beta)) + \lambda \|\beta\|_{L^1}, \quad \text{(S18)}$$

where λ is the tuning parameter for the L_1-penalty term. A 10-fold cross-validation was performed to select the optimal values of the tuning parameter for minimizing the mean cross-validation error. The R package "glmnet" was used to perform the LASSO method.

The nonzero components in the estimated $\hat{\beta}$ at the optimal tuning parameter were computed as risk coefficients, which were used to formularize a risk signature using the learning dataset. More precisely, we formulated the following risk score (RS) for each patient based on the expression levels of the selected genes: RS = (0.001695826 × ANPEP) + (0.001351164 × DPP4) + (0.828492221 × PRRG1) + (0.002693736 × GPNMB) + (0.572250065 × TMEM26) + (0.011112329 × PXDN) + (0.000861924 × CDH6) – (0.877296902 × SCN3A) – (0.042307865 × SEMA6B) + (0.019673956× CCDC37) + (1.184362541 × FANCA) + (0.101032334× NETO2).

The same risk signature was used to compute the risk scores for patients in the validation dataset. The patients in each dataset were classified into a high-risk group and a low-risk group according to the optimal cutoff value using the ROC method. The Kaplan–Meier (K–M) curves for patients in two groups were analyzed, and the statistical significance of the difference was assessed using the two-sided log-rank test. Time-dependent ROC analysis was further conducted to evaluate the prognostic accuracy of the above risk score with respect to the 3- and 5-year survival predictions of patients in both the learning and validation sets.

Targeted therapeutic response prediction

Survival and gene expression data for glioma patients who received targeted therapies in the validation set were extracted to evaluate the predictive effectiveness of the macrophage-related gene signature for classifying patients into drug-sensitive and drug-resistant groups. The 3- or 5-year survival status (alive or dead) was defined as the outcome (sensitive or resistant) of targeted drug treatment. The above risk signature was used to classify each patient into a sensitive group (i.e., low-risk group) or a resistant group (i.e., high-risk group) according to the optimal cutoff value of the risk score using the ROC method. To further compare the predictive power of different gene signatures for predicting the responses to the targeted therapy, we calculated the AUCs of the ROC curves to assess their accuracies.

Multivariate COX regression analysis and the combined signature

To assess whether the macrophage-related gene signature was independently correlated with the prognosis of glioma patients, we conducted univariate and multivariate COX regression analyses of clinicopathological factors and available gene signatures. Clinicopathological information, including age, gender and grade, was available for glioma patients in both the learning and validation sets. We also included the following gene signatures in the multivariate COX regression analyses: Signature 1: macrophage-related gene signature; Signature 2: EGFR gene signature; Signature 3: Cheng et al. signature.

The above risk factors that were significantly correlated with the overall survival and 5-year survival of glioma patients in both the learning and validation datasets were extracted to construct a combined signature using the LASSO method for variable selection. As a result, we defined the combined signature as follows: CS = (0.008974621 × Age) + (1.617859481 × Grade) + (0.940077644 × Signature_1) + (0.006408624 × Signature_3). Here, Grade = 1 for lower grade glioma, and Grade = 2 for high grade glioma.

We examined whether the combined signature could significantly improve the prognostic accuracy of the macrophage-related gene signature. The areas under time-dependent ROC curves were calculated to compare the prognostic accuracies of age, grade (low- or high-grade), signature 1 (i.e., the macrophage-related gene signature), signature 3 (i.e., Cheng et al. signature) and the combined signature.

Table S1. Scheme of sample grouping.

Grouping	Name of Sample	Name of Gene Chip
Reference sample	EP	49823_EP, 49825_EP, 49830_EP, 49831_EP, 49843_EP, 49845_EP
Pertubed samples	$EPUREB_1$	49823_EP, 49825_EP, 49830_EP, 49831_EP, 49843_EP, 49845_EP, 49041_Reb
	$EPUREB_2$	49823_EP, 49825_EP, 49830_EP, 49831_EP, 49843_EP, 49845_EP, 49059_Reb
	$EPUREB_3$	49823_EP, 49825_EP, 49830_EP, 49831_EP, 49843_EP, 49845_EP, 49827_Reb
	$EPUREB_4$	49823_EP, 49825_EP, 49830_EP, 49831_EP, 49843_EP, 49845_EP, 49849_Reb

Table S2. List of genes in the differential network.

Gene Symbol	Description
Fanca	Fanconi Anemia Complementation Group A
Ltc4s	Leukotriene C4 Synthase
Spsb4	SplA/Ryanodine Receptor Domain And SOCS Box Containing 4
Pxdn	Peroxidasin
Ptk7	Protein Tyrosine Kinase 7 (Inactive)
Cdh6	Cadherin 6
Wfdc1	WAP Four-Disulfide Core Domain 1
Col19a1	Collagen Type XIX Alpha 1 Chain
2810433D01Rik	
5033406O09Rik	
Ptger2	Prostaglandin E Receptor 2
Tanc1	Tetratricopeptide Repeat, Ankyrin Repeat And Coiled-Coil Containing 1
Cd24a	CD24 Antigen (Small Cell Lung Carcinoma Cluster 4 Antigen)
Tmem26	Transmembrane Protein 26
Gpr171	G Protein-Coupled Receptor 171
Btla	B And T Lymphocyte Associated
Scn3a	Sodium Voltage-Gated Channel Alpha Subunit 3
Lyz1	Lysozyme
Abcg4	ATP Binding Cassette Subfamily G Member 4
Arg1	Arginase 1
Sema6b	Semaphorin 6B
Pcdhb19	Protocadherin Beta 19 Pseudogene
Ptprf	Protein Tyrosine Phosphatase, Receptor Type F
BC068157	Proline Rich 36
Mip	Major Intrinsic Protein Of Lens Fiber
Neto2	Neuropilin And Tolloid Like 2
Dpp4	Dipeptidyl Peptidase 4
Gm11744	Photoreceptor Disc Component
Xcr1	X-C Motif Chemokine Receptor 1
Sh3tc2	SH3 Domain And Tetratricopeptide Repeats 2
Retnlg	Resistin Like Beta
Eps8l2	EPS8 Like 2
Mir1931	
Rep15	RAB15 Effector Protein
Cd4	CD4 Molecule
B330016D10Rik	
3110047P20Rik	NACHT And WD Repeat Domain Containing 2
Anpep	Alanyl Aminopeptidase, Membrane
Zfp811	
Prrg1	Proline Rich And Gla Domain 1
Mia1	Cilia And Flagella Associated Protein 100
Gpnmb	Glycoprotein Nmb

Table S3 Univariate Cox regression analysis for the association of each signature gene with the overall survival of glioma patients.

Symbol	Gene Name	Cells	HR (95%)	P value
**ANPEP**	Alanyl Aminopeptidase, Membrane	MΦ	1.043 (1.027-1.059)	2e-05
**DPP4**	Dipeptidyl Peptidase 4	MΦ	1.054 (1.030-1.079)	4e-04
**PRRG1**	Proline Rich And Gla Domain 1	MΦ	1542 (11.69-203497)	0.005
**GPNMB**	Glycoprotein Nmb	MΦ	1.068 (1.031-1.105)	0.003
**TMEM26**	Transmembrane Protein 26	MΦ	2.708 (2.088-3.511)	1e-09
**PXDN**	Peroxidasin	TC	1.032 (1.023-1.041)	2e-09
**CDH6**	Cadherin 6	TC	1.119 (1.079-1.161)	1e-07
**SCN3A**	Sodium Voltage-Gated Channel Alpha Subunit 3	TC	0.021 (0.003-0.158)	2e-05
**SEMA6B**	Semaphorin 6B	TC	0.915 (0.890-0.941)	1e-12
**CCDC37**	Cilia- and flagella-associated protein 100	TC	1.038 (1.022-1.054)	9e-04
**FANCA**	Fanconi anaemia, complementation group A	TC	6.616 (3.381-12.95)	3e-06
**NETO2**	Neuropilin And Tolloid Like 2	TC	2.049 (1.503-2.794)	9e-05

Table S5. Experimental evidences for functional roles of the 5 macrophage-related genes in cancer progression and/or drug resistance.

Gene	Functional roles in cancers	PMID	Refs
**ANPEP**	Silencing FLI or targeting CD13/ANPEP lead to dephosphorylation of EPHA2, a mediator of BRAF inhibitor resistance, and induce growth arrest or apoptosis in melanoma cells	29048432	[3]
	CD13-positive bone marrow-derived myeloid cells promote angiogenesis, tumor growth, and metastasis	24297924	[4]
**DPP4**	Dipeptidylpeptidase 4 inhibition enhances lymphocyte trafficking, improving both naturally occurring tumor immunity and immunotherapy	26075911	[5]
	DPP4 in anti-tumor immunity: going beyond the enzyme	26233333	[6]
	Suppression of lung metastases by the CD26/DPP4 inhibitor Vildagliptin in mice	26194276	[7]
**PRRG1**	Cancer development	26233958	[8]
**GPNMB**	Glioma-associated microglia/macrophages display an expression profile different from M1 and M2 polarization and highly express Gpnmb and Spp1	25658639	[9]
	Glycoprotein nonmetastatic melanoma protein B, a potential molecular therapeutic target in patients with glioblastoma multiforme	16609006	[10]
**TMEM26**	Expression of transmembrane protein 26 (TMEM26) in breast cancer and its association with drug response	27224909	[11]

The table lists the gene name, the biological functions in cancers and the corresponding PubMed IDs and literature references of each of the eight-macrophage-related genes.

Figure S1

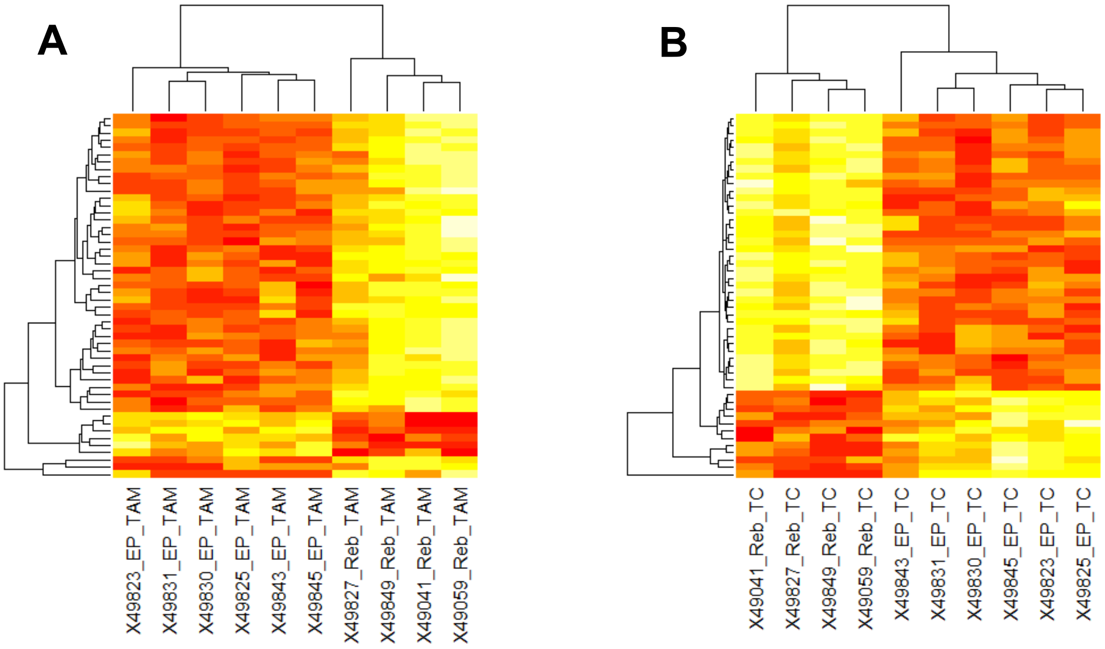

Figure S1. Heatmaps showing the hierarchical clustering of the differential gene expression profiles of TAMs (**A**) and TCs (**B**) between the Endpoint and Rebound groups.

Figure S2

A

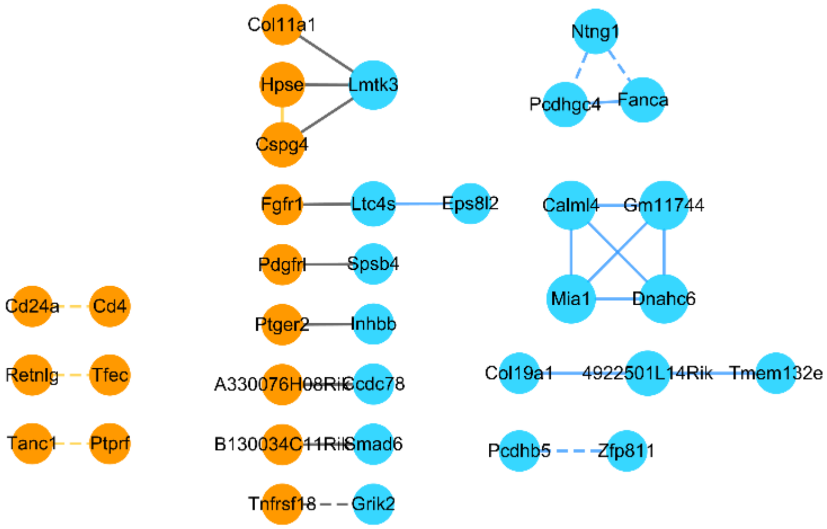

B

Number of nodes	35
Network diameter	2
Network centralization	0.047
Average number of neighbors	1.486

Figure S2. TC-TAM sensitive network and its topological attributes. (**A**) High-associated sensitive network. The node label represents the gene name. The size of the node represents its connectivity. The orange and blue nodes represent the genes in TAMs and TCs, respectively. High correlations (p-value less than 0.05, absolute value over 0.95) were utilized to construct the networks. The black edges represent the high correlations of gene pairs across TAMs and TCs; the orange and blue edges represent correlations of gene pairs within TAMs and TCs, respectively. The solid edge represents the positive correlation, and the dotted edge represents the negative correlation. (**B**) Topological attributes of the high-associated sensitive network.

Figure S3

A

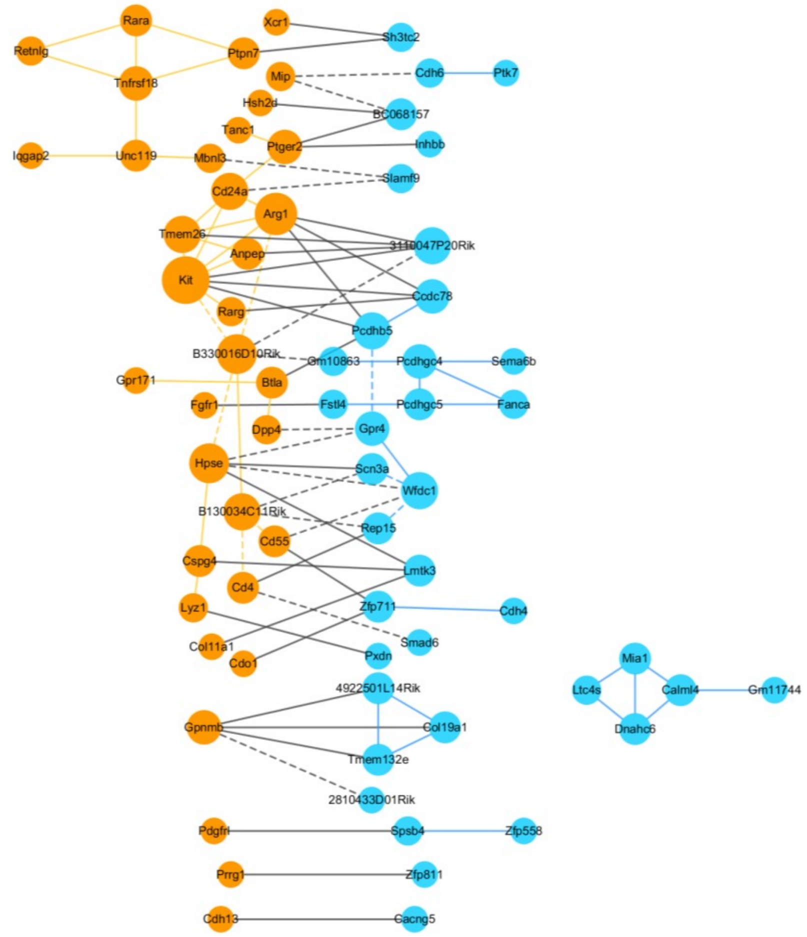

B

Number of nodes	72
Network diameter	14
Network centralization	0.092
Average number of neighbors	2.667

Figure S3. Ep U Reb_1 sample-specific TC-TAM perturbation network and its topological attributes. (**A**) High-associated sample-specific perturbation network. The node label represents the gene name. The size of the node represents its connectivity. The orange and blue nodes represent the genes in TAMs and TCs, respectively. High correlations (absolute value over 0.95, p-value less than 0.05) were utilized to construct the network. The black edges represent the high correlation of gene pairs across TAMs and TCs; the orange and blue edges represent correlations of gene pairs within TAMs and TCs, respectively. The solid edge represents the positive correlation, and the dotted edge represents the negative correlation. (**B**) Topological attributes of Ep U Reb_1 sample-specific TC-TAM perturbation networks.

Figure S4

A

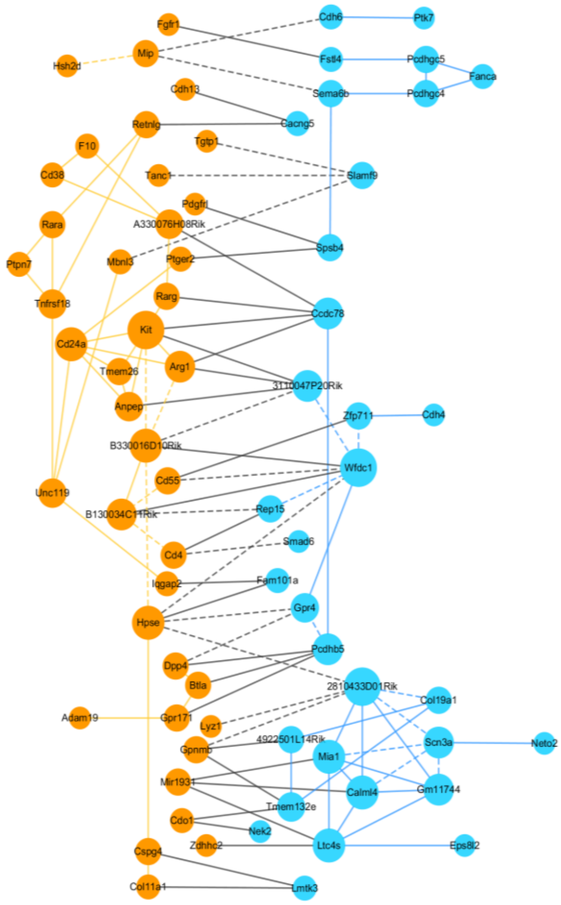

B

Number of nodes	73
Network diameter	15
Network centralization	0.070
Average number of neighbors	3.123

Figure S4. Ep U Reb$_2$ sample-specific TC-TAM perturbation network and its topological attributes. (**A**) High-associated sample-specific perturbation network. The node label represents the gene name. The size of the node represents its connectivity. The orange and blue nodes represent the genes in TAMs and TCs, respectively. High correlations (absolute value over 0.95, p-value less than 0.05) were utilized to construct the network. The black edges represent the high correlation of gene pairs across TAMs and TCs; the orange and blue edges represent correlations of gene pairs within TAMs and TCs, respectively. The solid edge represents the positive correlation, and the dotted edge represents the negative correlation. (**B**) Topological attributes of Ep U Reb$_2$ sample-specific TC-TAM perturbation networks.

Figure S5

A

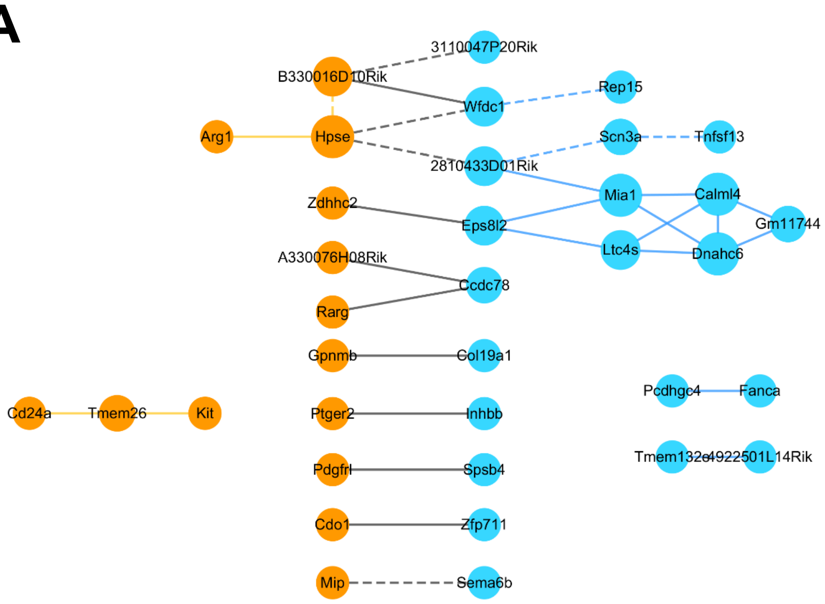

B

Number of nodes	36
Network diameter	6
Network centralization	0.069
Average number of neighbors	1.722

Figure S5. Ep U Reb$_3$ sample-specific TC-TAM perturbation network and its topological attributes. (**A**) High-associated sample-specific perturbation network. The node label represents the gene name. The size of the node represents its connectivity. The orange and blue nodes represent the genes in TAMs and TCs, respectively. High correlations (absolute value over 0.95, p-value less than 0.05) were utilized to construct the network. The black edges represent the high correlation of gene pairs across TAMs and TCs; the orange and blue edges represent correlations of gene pairs within TAMs and TCs, respectively. The solid edge represents the positive correlation, and the dotted edge represents the negative correlation. (**B**) Topological attributes of Ep U Reb$_3$ sample-specific TC-TAM perturbation networks.

Figure S6

A

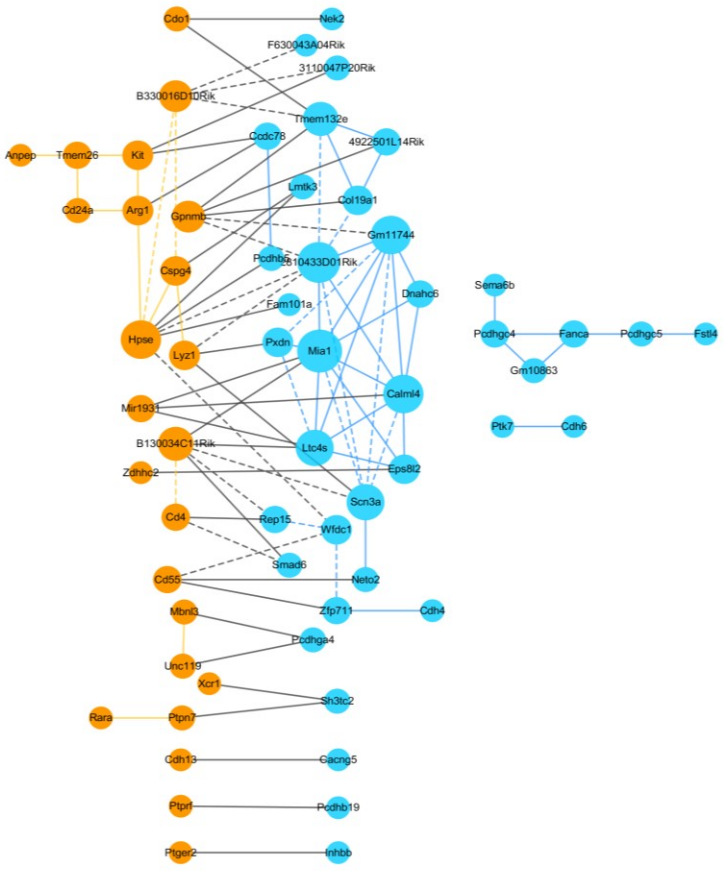

B

Number of nodes	62
Network diameter	8
Network centralization	0.117
Average number of neighbors	3.065

Figure S6. Ep U Reb_4 sample-specific TC-TAM perturbation network and its topological attributes. (**A**) High-associated sample-specific perturbation network. The node label represents the gene name. The size of the node represents its connectivity. The orange and blue nodes represent the genes in TAMs and TCs, respectively. High correlations (absolute value over 0.95, p-value less than 0.05) were utilized to construct the network. The black edges represent the high correlation of gene pairs across TAMs and TCs; the orange and blue edges represent correlations of gene pairs within TAMs and TCs, respectively. The solid edge represents the positive correlation, and the dotted edge represents the negative correlation. (**B**) Topological attributes of Ep U Reb_4 sample-specific TC-TAM perturbation networks.

Figure S7

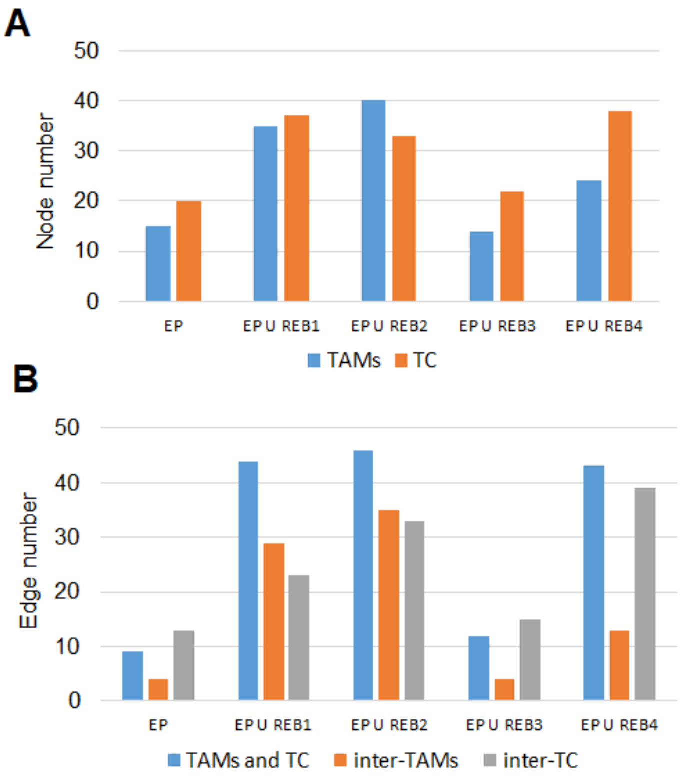

Figure S7. Network topological analysis of the sensitive network and 4 sample-specific perturbation networks. Node numbers (**A**) and edge numbers (**B**) of the sensitive network (Ep) and 4 sample-specific perturbation networks (EpUReb$_i$, i=1, 2, 3, 4).

Figure S8

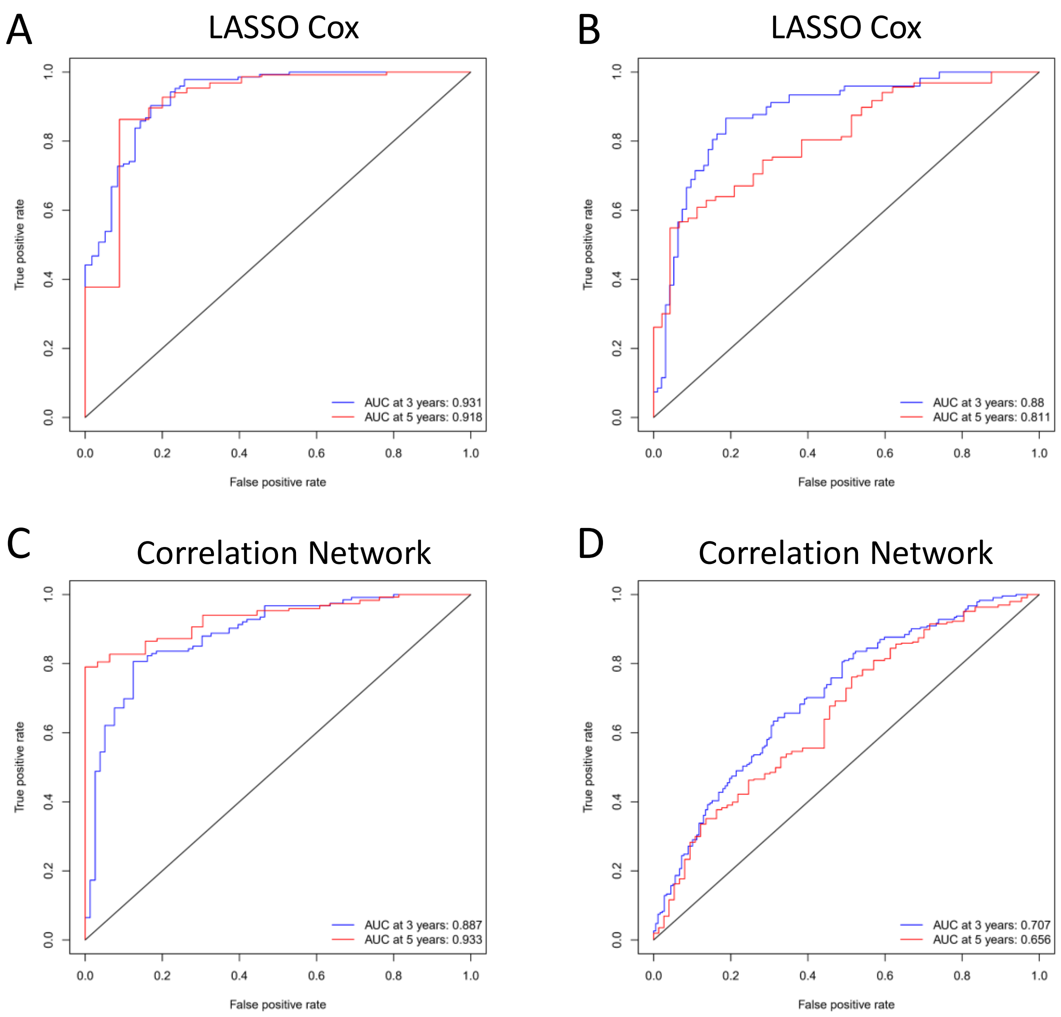

Figure S8. Prognostic accuracies of LASSO Cox signature and correlation network-based signature. (**A-B**) Time-dependent ROC curves of the LASSO Cox signature evaluated on the learning set (A) and the test set (B). (**C-D**) Time-dependent ROC curves of the correlation network-based signature evaluated on the learning set (C) and the test set (D).

Supplementary references

1. Quail DF, Bowman RL, Akkari L, Quick ML, Schuhmacher AJ, Huse JT, et al. The tumor microenvironment underlies acquired resistance to CSF-1R inhibition in gliomas. Science. 2016;352(6288).
2. Liu X, Wang Y, Ji H, Aihara K, Chen L. Personalized characterization of diseases using sample-specific networks. Nucleic Acids Research. 2016;44(22):e164.
3. Azimi A, Tuominen R, Costa Svedman F, Caramuta S, Pernemalm M, Frostvik Stolt M, et al. Silencing FLI or targeting CD13/ANPEP lead to dephosphorylation of EPHA2, a mediator of BRAF inhibitor resistance, and induce growth arrest or apoptosis in melanoma cells. Cell Death &Amp; Disease. 2017;8:e3029. doi: 10.1038/cddis.2017.406
4. Eleonora D, Roberto R, Liliana GR, Barbu EM, Hitomi H, John LS, St, et al. CD13-positive bone marrow-derived myeloid cells promote angiogenesis, tumor growth, and metastasis. Proceedings of the National Academy of Sciences of the United States of America. 2013;110(51):20717-22.
5. Barreira da Silva R, Laird ME, Yatim N, Fiette L, Ingersoll MA, Albert ML. Dipeptidylpeptidase 4 inhibition enhances lymphocyte trafficking, improving both naturally occurring tumor immunity and immunotherapy. Nature Immunology. 2015;16:850. doi: 10.1038/ni.3201
6. Jang JH, Baerts L, Waumans Y, Meester ID, Yamada Y, Limani P, et al. Suppression of lung metastases by the CD26/DPP4 inhibitor Vildagliptin in mice. Clinical & Experimental Metastasis. 2015;32(7):677-87.
7. Ohnuma K, Hatano R, Morimoto C. DPP4 in anti-tumor immunity: going beyond the enzyme. Nature Immunology. 2015;16:791. doi: 10.1038/ni.3210.
8. Hanqing Z, Chang H, Tong W. MiR-17-92 cluster promotes hepatocarcinogenesis. Carcinogenesis. 2015;36(10):1213-22.
9. Szulzewsky F, Pelz A, Feng X, Synowitz M, Markovic D, Langmann T, et al. Glioma-Associated Microglia/Macrophages Display an Expression Profile Different from M1 and M2 Polarization and Highly Express Gpnmb and Spp1. Plos One. 2015;10(2):e0116644.
10. Kuan CT, Wakiya K, Dowell JM, Herndon NJ, Reardon DA, Graner MW, et al. Glycoprotein nonmetastatic melanoma protein B, a potential molecular therapeutic target in patients with glioblastoma multiforme. Clinical Cancer Research An Official Journal of the American Association for Cancer Research. 2006;12(7 Pt 1):1970.
11. Nass N, Dittmer A, Hellwig V, Lange T, Mirjam BJ, Leyh B, et al. Expression of transmembrane protein 26 (TMEM26) in breast cancer and its association with drug response. Oncotarget. 2016;7(25):38408-26.
